# Ten Years of Artificial Intelligence in Screening Mammography: A Systematic Review and Meta-Analysis of Diagnostic Accuracy and Clinical Implementation (Literature Published 2015–2025)

**DOI:** 10.3390/diagnostics16183045

**Published:** 2026-09-20

**Authors:** Sebastian Ciurescu, Victor Buciu, Diana-Gabriela Ilaș, Raluca Pârvănescu, Denis Șerban

**Affiliations:** 1 Doctoral School in Medicine, Victor Babeș University of Medicine and Pharmacy, 300041 Timisoara, Romania; sebastian.ciurescu@umft.ro (S.C.); raluca.reitmayer@umft.ro (R.P.); 2 Department of Medical Semiology, Victor Babeș University of Medicine and Pharmacy, 300041 Timisoara, Romania; 3 Department of Obstetrics and Gynecology, Victor Babeș University of Medicine and Pharmacy, 300041 Timisoara, Romania; denis.serban@umft.ro

**Keywords:** artificial intelligence, deep learning, mammography, breast-cancer screening, diagnostic accuracy, meta-analysis, computer-aided detection

## Abstract

**Background:** Deep-learning artificial intelligence (AI) for mammographic screening moved from proof of concept to randomised evaluation in a single decade. We reviewed and meta-analysed its diagnostic accuracy and its effect on screening programmes, covering the literature published between 2015 and 2025. **Methods:** PubMed and Europe PMC were searched from 1 January 2015 to 31 December 2025, supplemented by ClinicalTrials.gov and by forward and backward citation searching (PRISMA 2020, PRISMA-DTA, PRISMA-S). Eligible studies evaluated a deep-learning system for cancer detection or triage in a screening population against a histopathological reference standard. Four syntheses were performed: standalone accuracy pooled on the logit-AUC scale (A), a bivariate sensitivity–specificity model (A2), cancer detection rate ratio for AI-integrated versus standard reading (B), and recall rate ratio (C). Random-effects models used restricted maximum likelihood with Knapp–Hartung intervals. Risk of bias was assessed with QUADAS-2 and QUADAS-C, and certainty with GRADE. **Results:** Twenty-six studies (27 reports, 2019–2025) were included. Pooled standalone AUC across 14 studies and 1,214,885 examinations was 0.890 (95% CI 0.858–0.915), with I^2^ = 97.4% and a 95% prediction interval of 0.731–0.960. Neither publication year (*p* = 0.79) nor enriched versus consecutive sampling (*p* = 0.94) explained this dispersion in meta-regression. The bivariate model (k = 9) gave a summary sensitivity of 73.3% (64.5–80.6) at a specificity of 92.4% (86.8–95.8). Across randomised and paired prospective trials (k = 3), the pooled detection rate ratio was 1.13 (0.83–1.55), with the MASAI randomised trial alone reporting 1.29 (1.09–1.51) and a 44% reduction in screen reading. Non-randomised implementation studies (k = 5), which include a 463,094-women German programme evaluation, pooled to 1.22 (1.08–1.37) with little dispersion (I^2^ = 19.0%). Recall changed little overall (0.95, 0.81–1.12). Certainty was very low for accuracy outcomes and moderate for the single randomised trial. **Conclusions:** A decade of evidence supports AI as a second reader and triage tool in organised screening, not as an autonomous replacement for the radiologist. Pooled accuracy is high on average but so dispersed that it cannot be transferred to a new programme; local validation before deployment remains necessary. The larger and more precise detection gains come from non-randomised designs, which is the pattern confounding would produce, so the randomised evidence remains the anchor. Interval-cancer and mortality endpoints are still awaited.

## 1. Introduction

Breast cancer is the most frequently diagnosed cancer and a leading cause of cancer death in women worldwide, with an estimated 2.3 million new cases in 2020 [1]. Population-based mammographic screening reduces breast-cancer mortality. Systematic reviews prepared for the European Commission Initiative on Breast Cancer and for the Canadian Task Force on Preventive Health Care, together with the evaluation of the IARC Working Group, place the relative mortality reduction on the order of 20–40% among invited women [2,3,4], although the magnitude and the balance of benefits and harms remain debated, and the methodological limitations of some historical trials have themselves been the subject of scientific review [5]. These constraints motivate continued innovation in how screening images are interpreted.

Two limitations of conventional interpretation are persistent. Sensitivity falls in radiographically dense breasts, where cancers may be masked by overlapping fibroglandular tissue. Dense breasts are associated with later-stage disease at diagnosis and a higher rate of interval cancers, and quantitative density and masking-risk tools have been developed to identify the women most affected [6,7,8,9]. Separately, screening programmes face a growing mismatch between rising examination volumes and a limited breast-radiology workforce, an access problem that is especially acute in lower-resource settings [10]. Double reading, the standard of care in most European programmes, improves cancer detection but roughly doubles the reading workload.

Computer-aided detection systems introduced in the early 2000s aimed to ease these pressures. They were limited by high false-mark rates and, in a large community-practice analysis, failed to improve, and for some measures worsened, screening accuracy [11]. The deep-learning methods that emerged in the mid-2010s were built on convolutional neural networks and later transformer architectures trained on large annotated datasets. They produced a qualitatively different generation of tools that learn image features directly rather than relying on hand-engineered descriptors [12].

Our group has contributed to this transition in two ways: methodologically, by developing and validating a ResNet50-based classifier for two-dimensional mammography that reached an AUC of 0.93 in a single-centre cohort [13], and through evidence evaluation of AI diagnostic performance alongside inflammation-based prognostic markers [14]. The present review widens the lens from a single algorithm and a single-centre dataset to the full decade of screening-relevant evidence.

Between 2015 and 2025, the field moved through a recognisable arc. Retrospective reader studies showed that standalone AI could match average radiologist accuracy. External-validation work then tested performance on independent, population-based cohorts [15,16]. Prospective paired-reader trials replaced one human reader with AI at scale, and, by 2023, a randomised trial had reported a pre-specified endpoint for cancer detection.

Four previous syntheses bracket this period [17,18,19,20], and a fifth appeared while this review was in preparation [21]. Each of them answered a cross-sectional question: how accurate is AI on average, at the time of writing. None of them could answer the question that now matters to a programme director, because the relevant trials had not reported.

What this review adds. Three things distinguish this work from the earlier syntheses and from our own 2025 review [14]. First, it treats the design of the evidence as an object of analysis rather than a nuisance, testing formally whether reported accuracy has improved over the decade instead of asserting that it has. Second, it separates standalone accuracy from programme-level effect throughout, and it refuses to pool implementation studies whose comparators differ, presenting them by evidence tier instead. Third, every estimate reported here is regenerated by a version-controlled script from extraction tables that are published with the article, so that any reader can re-run the synthesis when the next trial reports.

Review question. Framed with the PIRD structure: in women undergoing screening mammography (Population), how does a deep-learning AI system used standalone or integrated with radiologist reading (Index test) perform for the detection of screen-relevant breast cancer (Diagnosis/Target condition), verified against histopathology with cancer-free follow-up (Reference standard), relative to standard radiologist readings? The primary endpoints were the area under the ROC curve and paired sensitivity and specificity for standalone accuracy, and the cancer detection rate ratio and recall rate ratio for AI-integrated reading.

A note on the date range. The title refers to the decade of literature that was searched: 1 January 2015 to 31 December 2025. No study meeting the eligibility criteria was published before 2019, so the included studies span 2019–2025. That gap is itself a finding, and it is why the review is framed around a decade of evidence rather than a decade of publications.

## 2. Materials and Methods

### 2.1. Protocol and Registration

The review followed the PRISMA 2020 [22], PRISMA-DTA [23] and PRISMA-S [24] statements. The completed PRISMA 2020 and PRISMA-DTA checklists are provided as Tables S6 and S7. The protocol and the data-extraction form were deposited on protocols.io (https://dx.doi.org/10.17504/protocols.io.q26g7q469lwz/v1) before screening was completed and are reproduced as Protocol S1. Deviations from the protocol are listed in Section 2.9.

### 2.2. Eligibility Criteria

Eligibility followed the PIRD framework.

Population. Women undergoing mammographic screening, either digital mammography or digital breast tomosynthesis. Studies confined to symptomatic or diagnostic populations were excluded.

Index test. A deep-learning system used standalone or integrated with radiologist reading, whether as triage, second reader or reader replacement, producing an examination-level or lesion-level suspicion score.

Reference standard. Histopathology for screen-detected and clinically detected cancers, with a cancer-free follow-up interval of at least 12 months for negative examinations.

Target condition. Screen-relevant breast cancer, invasive or in situ.

Eligible designs were multi-reader multi-case studies, retrospective consecutive or case–control accuracy studies, external-validation studies, prospective paired-reader studies, before-and-after programme comparisons and randomised controlled trials. We excluded reviews, editorials and protocols without primary data; non-mammographic modalities; studies of pure risk prediction without a detection or triage endpoint; and simulation or decision-modelling studies without a real reading comparison. Development studies evaluated only on public benchmark collections were excluded because they have no screening population and no follow-up-based reference standard.

Publications in languages other than English were not eligible, because the review team could not appraise full texts reliably without translation and no translation budget was available. Twenty-four non-English records were identified. Their titles and English abstracts were inspected, and none described a screening-population evaluation that was not also reported in English; the exclusion is nonetheless recorded as a limitation.

The grey literature was searched rather than assumed absent. Preprints indexed in Europe PMC and trial-registry records in ClinicalTrials.gov were screened under the same criteria. Conference abstracts were eligible only if they reported extractable accuracy or programme outcomes; none did.

### 2.3. Information Sources and Search

PubMed and Europe PMC were searched on 11 August 2026 for records published between 1 January 2015 and 31 December 2025. ClinicalTrials.gov was searched on the same date for completed and ongoing evaluations. Reference lists of all included studies and of the five previous systematic reviews [17,18,19,20,21] were then searched forward and backward through the Europe PMC citation network.

The PubMed strategy combined controlled vocabulary and free text across three concept blocks (artificial intelligence or deep learning; mammography or tomosynthesis; screening or diagnostic accuracy), restricted to human studies published in English. The complete line-by-line strategy for every source, with the yield at each step, is given in Table S1.

The strategy reported in our original submission contained an error that a reviewer identified: it used the MeSH term “Tomography, X-Ray Computed”, which indexes computed tomography rather than breast tomosynthesis, and it contained no free-text term for tomosynthesis or DBT. That strategy returned 4043 records, inflated by the computed-tomography literature while missing tomosynthesis-specific reports. The corrected strategy used here removes the computed-tomography term and adds mammograph*[tiab], tomosynthes*[tiab], "digital breast tomosynthesis"[tiab] and "Breast Neoplasms/diagnostic imaging"[MeSH]. All screening and extraction were repeated from the corrected set.

Embase, Scopus, Web of Science, IEEE Xplore and the Cochrane Library were not searched. None is available under our institutional subscriptions, and none offers a programmatic interface through which a search could be documented reproducibly.

### 2.4. Study Selection

Records were exported with their identifiers and deduplicated automatically, first on DOI, then on PubMed identifier, then on a normalised title string; 674 duplicates were removed. The deduplicated set of 1743 records was screened on title and abstract against a written rule set that operationalised each eligibility criterion (Section S1). Every exclusion was logged with its reason, and the full screening log is published with the data package so that the first pass can be audited or re-run.

All 151 records that survived the first pass were retrieved in full and assessed independently in duplicate by two reviewers (S.C., R.P.). Disagreements were resolved by discussion and, where discussion did not settle the question, by a third reviewer (V.B.). A further six reports were reinstated after the first pass and assessed the same way: three recovered from reference lists at the first revision and three by the screening verification described below, giving 157 reports assessed for eligibility. Every inclusion decision in this review therefore carries the agreement of at least two assessors.

Because a single deterministic rule set provides no independent check on the first pass, at the second revision we verified that stage against a second, independently specified screening procedure applied to all 1743 records. The two procedures differ in both information source and logic: the first works from the free text of the title and abstract through a sequential exclusion cascade, the second from NLM Medical Subject Headings and author keywords through additive inclusion scoring across the four PIRD concepts, with free text used only where indexing terms are absent. The rule sets were written separately and the second was not tuned to reproduce the first. The full detail, the comparison and the complete discordance listing are in Section S1, and the code is in Data S1.

Agreement between the two procedures was moderate: Cohen’s κ 0.47 (95% CI 0.41–0.53) with 85.7% raw agreement. The second procedure was markedly more inclusive, carrying 385 records to full text against 151, and all 250 discordant records were adjudicated in two stages. Every record was first assessed against the eligibility criteria of Section 2.2 at record level, using its title, abstract and indexing terms; 230 failed a criterion unambiguously and are recorded as ineligible with the criterion each fails. The 20 records with any plausible route to inclusion were then retrieved in full and adjudicated against the complete criteria by two reviewers, with disagreements resolved by discussion. The first stage is a single documented assessment rather than duplicate independent human screening, and we do not present it as more than that. The complete 250-record adjudication, with the decision, the reason and the stage that decided each record, is screening_adjudication.csv in Data S1, and Section S1.6 summarises it. The exercise identified three eligible studies the first pass had missed, all of which are now included, and confirmed that a further three studies missed by the first pass were already in the review because they had been retrieved by citation searching. Section 3.1 reports the outcome and Section 4.1 states what it implies about the completeness of the first pass.

For the full-text stage, the duplicate assessments were reconciled to consensus as they were made rather than being recorded separately beforehand, so the disagreement counts a kappa requires were not preserved for that stage.

### 2.5. Data Extraction

This section describes how study-level data were transferred onto the extraction sheet. It is not a description of study selection, which is covered in Section 2.4, nor of the statistical models, which are covered in Section 2.7.

Two reviewers independently extracted, onto a piloted form: study identifiers and publication year; country and screening programme; design; enrolment period; number of examinations and number of cancers; the AI system, its vendor and its version; the rule by which an operating threshold was chosen; the reference standard and its follow-up window; the comparator; and the outcome data. Outcome data comprised the AUC with its 95% confidence interval, sensitivity and specificity with the counts needed to reconstruct a 2 × 2 table, and, for implementation studies, cancer detection rate, recall rate, positive predictive value and screen-reading workload. Discrepancies were reconciled against the source report. The completed extraction tables, with one row per study with its PubMed identifier and DOI, are published as machine-readable files and reproduced as Table S4a–d. The blank extraction template is Table S8.

Several reports offer more than one candidate estimate, so two selection rules were needed. Neither was in the registered protocol. Both were written in response to peer review, before the affected studies were re-extracted, and applied uniformly from that point; they are amendments, not pre-specifications, and Section 2.9 records them as such.

1. Multiple AI systems in one study. Where a study evaluated several AI systems without nominating one as primary, the median-performing system entered the main analysis, with the best- and worst-performing systems carried into a sensitivity analysis. Taking the best performer, as our original submission did for Salim et al. [25], selects on the outcome and biases the pooled estimate upwards. Under the median rule, that study contributes AI-2, AUC 0.922 (0.910–0.934), rather than AI-1, AUC 0.956 (0.948–0.965).
2. Multiple cohorts in one study. Where a study reported several independent cohorts, the largest entered the main analysis and the others were carried into a sensitivity analysis. Schaffter et al. [26] therefore contributes the US cohort (144,231 examinations, AUC 0.858) rather than the Swedish cohort (AUC 0.903).

### 2.6. Risk of Bias and Certainty of Evidence

QUADAS-2 [27] was applied to all included studies across the four risk-of-bias domains and the three applicability domains, with QUADAS-C [28] used where a study made a comparative accuracy claim. Each study was assessed independently by two reviewers and disagreements were resolved by discussion. Signalling-question responses and the written rationale for every judgement are given in Table S2, and the traffic-light and domain summaries are reported in the Results.

TRIPOD+AI [29] is a reporting guideline, not a risk-of-bias instrument, and our original submission described it incorrectly as one. It is used here only to comment on the completeness of reporting. Where a study also developed the model it evaluated, PROBAST+AI [30] informed the index-test judgement.

Certainty of evidence was rated with GRADE for diagnostic test accuracy [31,32] for each synthesis outcome, across risk of bias, indirectness, inconsistency, imprecision and publication bias. The full evidence profile, with the reason recorded for every downgrade, is Table S3.

### 2.7. Statistical Analysis

Analyses were implemented in Python 3 (numpy, pandas, scipy) and the complete script is published with the article. Re-running it regenerates every number in this manuscript and every figure from the extraction tables; no effect estimate is typed by hand into the text or into the plotting code. A large language model (Claude, Anthropic) was used only for language editing of the manuscript text; it was not used for study selection, data extraction, statistical analysis or interpretation, and all edited text was checked by the authors.

Which studies entered which analysis. A study contributed to Analysis A if it reported an AUC for standalone AI in a screening population, either with a 95% confidence interval or with the case and control counts needed to approximate a standard error by the method of Hanley and McNeil [33]. It contributed to Analysis A2 if sensitivity, specificity and the corresponding denominators allowed a 2 × 2 table to be reconstructed. It contributed to Analysis B if it compared cancer detection rates between an AI-integrated reading arm and a standard-care arm with denominators for both, and to Analysis C if it reported recall rates for both arms. A study could contribute to more than one analysis; several contributed to none, and Table 1 states for each study exactly which analyses it entered and why.

Choice of outcome measure, and its limits. We report AUC as the primary standalone measure and sensitivity with specificity as a co-primary measure, and we would encourage readers to weight the second more heavily. AUC has real advantages for this literature: it is threshold-independent, so it can compare systems whose operating points were set by different vendors on different scales, and it is the measure most consistently reported, which is why a synthesis restricted to sensitivity and specificity would have covered fewer than half as many examinations. However, AUC is a poor summary of what a screening programme is actually trying to do. A screening test needs high sensitivity, because a missed cancer presents later and worse, together with a specificity high enough that the false-positive burden (recall, further imaging, biopsy, anxiety, cost) stays acceptable. AUC averages performance across every threshold, including regions of the ROC curve that no screening programme would ever operate in, and it can be identical for two systems with very different behaviours at the operating point that matters. It also says nothing about cost, uptake, or whether detecting a given cancer earlier changes what happens to the woman. For those reasons, the pooled AUC in Section 3.3 is presented as an exploratory summary of what has been reported, and the bivariate model in Section 3.4, the programme-level outcomes in Section 3.5 and the certainty ratings in Section 3.7 carry the interpretive weight.

Analysis A—standalone accuracy. Per-study AUCs were transformed to the logit scale before pooling, with standard errors transferred by the delta method. Pooling on the raw AUC scale, as our original submission did, allows a study with a very narrow interval to dominate and can produce intervals that stray outside the unit interval; the logit scale avoids both problems. Between-study variance was estimated by restricted maximum likelihood and confidence intervals were computed with the Knapp–Hartung–Sidik–Jonkman correction. This choice replaces the DerSimonian–Laird estimator used in the original submission. DerSimonian–Laird is the historical default, but it is known to underestimate the between-study variance when heterogeneity is substantial and to produce confidence intervals that are too narrow, particularly at small-to-moderate numbers of studies [57,58]. Both DerSimonian–Laird and the Paule–Mandel estimator were run as planned sensitivity analyses and are reported alongside the primary result. Heterogeneity was quantified with Cochran’s Q, I^2^ and τ^2^, and a 95% prediction interval was calculated to convey the range within which the accuracy of a future study would be expected to fall [59]. Leave-one-out analysis was performed for every study.

Exploring heterogeneity. Subgroups were specified before the analyses were run, though after the protocol was registered, by study design (enriched case–control or multi-reader sampling versus consecutive population cohort) and by region, with a minimum of three studies required for a subgroup to be analysed. Random-effects meta-regression was fitted with publication year, design and both together as covariates, with Knapp–Hartung standard errors, and R^2^ was reported as the proportion of between-study variance explained. Cohort overlap was handled by the rule in Section 2.8 and checked in the leave-one-out analysis.

Analysis A2—bivariate model. Logit sensitivity and logit specificity were modelled jointly in a bivariate random-effects model of the Reitsma type [60], estimated by maximum likelihood, giving a summary point in ROC space, the between-study standard deviations, their correlation and a summary diagnostic odds ratio. A full hierarchical summary ROC curve was not fitted. Fitting one requires several thresholds per study, and every study here reports a single operating point, chosen under a rule that differs between studies. The bivariate model is the appropriate reduction in that situation, and the reason each study’s threshold was chosen is tabulated alongside its estimate so that the reader can judge how comparable they are.

Analyses B and C—programme-level outcomes. Cancer detection rate ratios and recall rate ratios were pooled on the log scale with the same estimator. Studies were stratified by evidence tier before pooling: randomised or paired prospective trials in one tier, non-randomised implementation studies in the other. The two tiers were not combined. Within the prospective tier, the individual trials are reported as the primary result and the pooled estimate is secondary, because MASAI and ScreenTrustCAD tested different things: MASAI compared AI-triaged reading against standard double reading in a randomised parallel-group design, while ScreenTrustCAD replaced one of two radiologists with AI in a paired design applied to the same women. Where a trial reported an interval that accounted for the pairing, that interval was used; otherwise, intervals were derived from independent Poisson variances, which is conservative.

Small-study effects. With 14 studies, Analysis A meets the usual ten-study minimum for a test of funnel-plot asymmetry, so Egger’s regression was performed and the funnel plot is shown as Figure S2. Analyses A2, B and C each contain fewer than ten studies in every stratum, and no test was attempted for those; the tests are known to be badly underpowered at that size and to give misleading results in diagnostic accuracy reviews in particular [61].

### 2.8. Cohort Overlap

Several reports draw on shared screening infrastructure, and double counting the same women would give a false impression of precision. Six overlaps were identified and handled as follows. The MASAI interim safety analysis [62] and the MASAI primary screening-performance analysis [50] are the same trial; they are counted as one study and the primary-performance record is used for pooling. Two Danish reports [45,63] evaluate the same Region of southern Denmark cohort over the same period; only one is used in any single synthesis, and the more complete report [45] enters Analysis A2. Salim et al. [25] and ScreenTrustCAD [48] both recruit from Stockholm screening services but at different hospitals and in different periods; they enter different analyses and are therefore never pooled together, and the leave-one-out analysis confirms that omitting the earlier study does not change the conclusion.

Three further overlaps were identified at the second revision. A 2025 evaluation of two deep-learning models on 129,434 BreastScreen Norway examinations [64] draws on the same national programme as Larsen et al. [43]; the larger cohort was retained, and the later report is discussed narratively. A 2019 workload study uses the same 2652 examinations and 101 readers as the multi-reader study already included [34], and was excluded on that basis. Finally, the two United States reports of tomosynthesis implementation [49,54] share analytic collaborators and may draw on overlapping practices; because this could not be resolved from the published reports, both were retained and a sensitivity analysis omitting each in turn is reported in Section 3.5.

### 2.9. Deviations from the Protocol

The registered protocol specified the eligibility criteria, the information sources, the risk-of-bias and certainty tools, and Analyses A and B. Everything listed below was decided after the protocol was registered, and none of it should be read as pre-specified.

After the first round of peer review. The search was extended beyond PubMed to Europe PMC, ClinicalTrials.gov and citation searching, and the PubMed strategy was corrected as described in Section 2.3. The primary estimator was changed from DerSimonian–Laird on the AUC scale to restricted maximum likelihood with Knapp–Hartung intervals on the logit-AUC scale. The two selection rules in Section 2.5 were added. Analyses A2 and C were added. Subgroup and meta-regression analyses were added.

After the second round of peer review. The title-and-abstract screen was verified against an independently specified second procedure (Section 2.4), which led to three further studies being included. The evidence tiers in Analysis B were renamed to distinguish randomised and paired prospective designs from non-randomised implementation studies, and a prospective non-randomised programme evaluation was assigned to the latter. Sensitivity analyses for the two newly identified cohort overlaps were added.

## 3. Results

### 3.1. Study Selection

**Figure 1:**
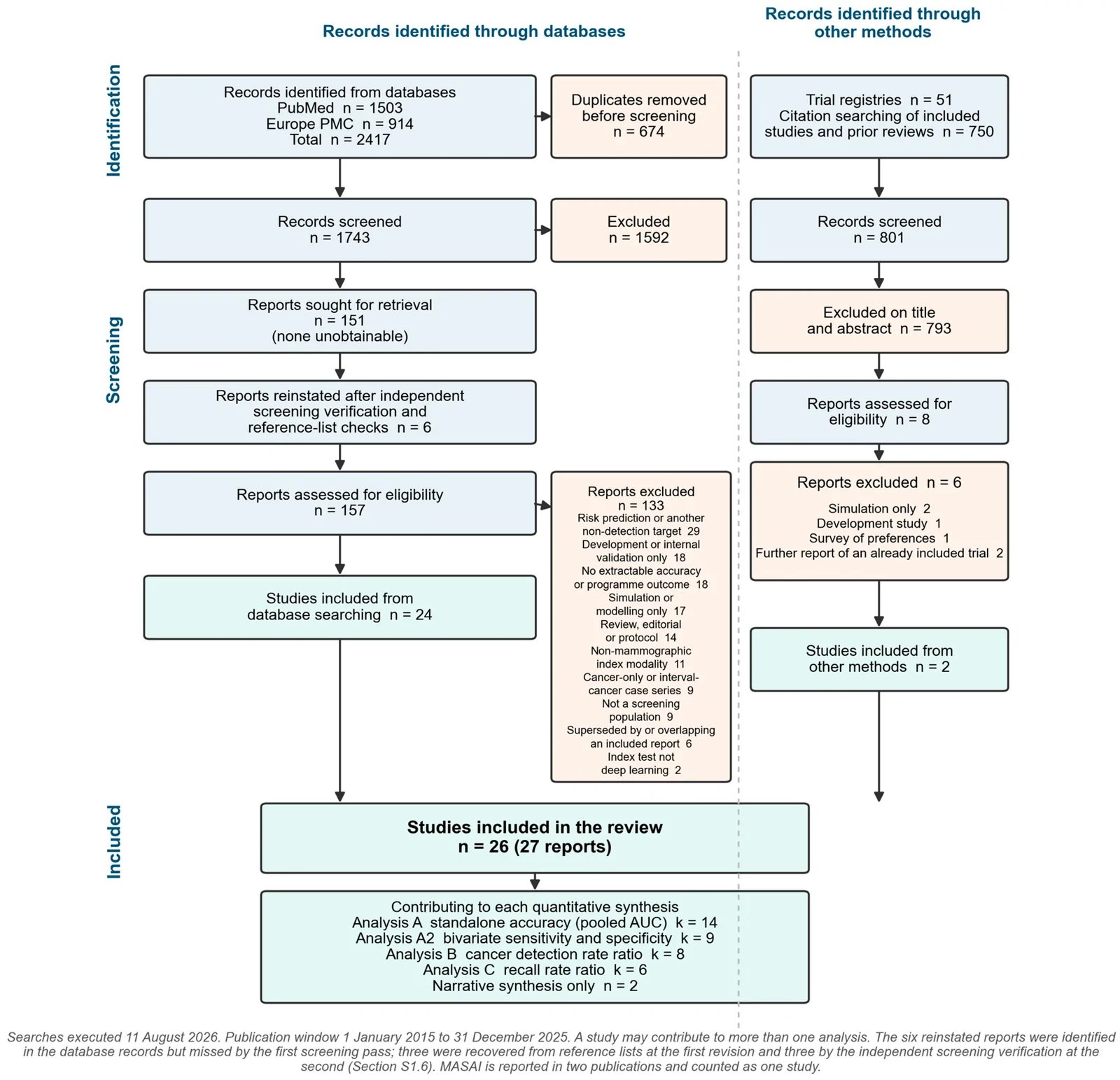
PRISMA 2020 flow diagram for the review, showing database and non-database sources separately, with itemised reasons for exclusion at full-text assessment.

The corrected searches returned 1503 records from PubMed and 914 from Europe PMC. After 674 duplicates were removed, 1743 records were screened on title and abstract and 1592 were excluded. All 151 remaining reports were retrieved, and six further reports that the first pass had missed were reinstated (Section 2.4), giving 157 reports assessed in full, of which 133 were excluded and 24 included. The reasons are itemised in Figure 1 and listed report by report in fulltext_exclusions.csv in Data S1; the commonest were that the study addressed future-risk prediction or another non-detection target (*n* = 29), was a development or internal-validation study without an independent screening cohort (*n* = 18), or reported no accuracy or programme outcome the syntheses could use (*n* = 18). Searching outside the databases contributed 801 further records (51 trial-registry entries and 750 unique records from the citation network), of which eight were assessed in full and two were included [45,48].

Independent verification of the title-and-abstract screen (Section 2.4) changed the included set. The second screening procedure flagged 242 records that the first had excluded, and adjudication of all 250 discordant records identified three eligible studies the first pass had missed: a nationwide German implementation study of 463,094 women [55], a multicentre multivendor validation of a triage algorithm [38], and a site-controlled evaluation of tomosynthesis screening with AI [49]. All three are now included. The same exercise showed that three studies already in the review [34,47,51] had also been missed by the first pass and had entered only through citation searching, which puts the sensitivity of that pass at 18 of the 24 eligible studies present in the record set, or 75%. The remaining 244 records were adjudicated ineligible. Two of them were eligible on design but excluded under the cohort-overlap rule [64]; the criterion behind every other decision is recorded in the adjudication file, and Section S1.6 summarises them.

Twenty-six studies, reported in 27 publications, met the criteria (Figure 1); MASAI is the only study reported twice. Fourteen contributed to Analysis A, nine to Analysis A2, eight to Analysis B and six to Analysis C. Two studies contributed to the narrative synthesis only: one because the denominators for its year-specific detection rates were not separately reported [56], the other because cancer counts were not reported in a form that could be pooled [15]. Table 1 states the position of every included study.

### 3.2. Characteristics of Included Studies

The included studies were published between 2019 and 2025 and were conducted in 14 countries across Europe, North America, Asia, Oceania and the eastern Mediterranean. Enrolment periods ran from 2004 to 2023. Eleven commercial systems and four academic systems were evaluated; Transpara, Lunit INSIGHT MMG and iCAD each appeared in more than one study. Twenty-four studies evaluated a system on data independent of its development.

Table 1 gives the characteristics of all 26 studies and states which analyses each entered. The “Examinations” column reports screening examinations analysed; for the implementation studies, the two arms are summed.

**Figure 2:**
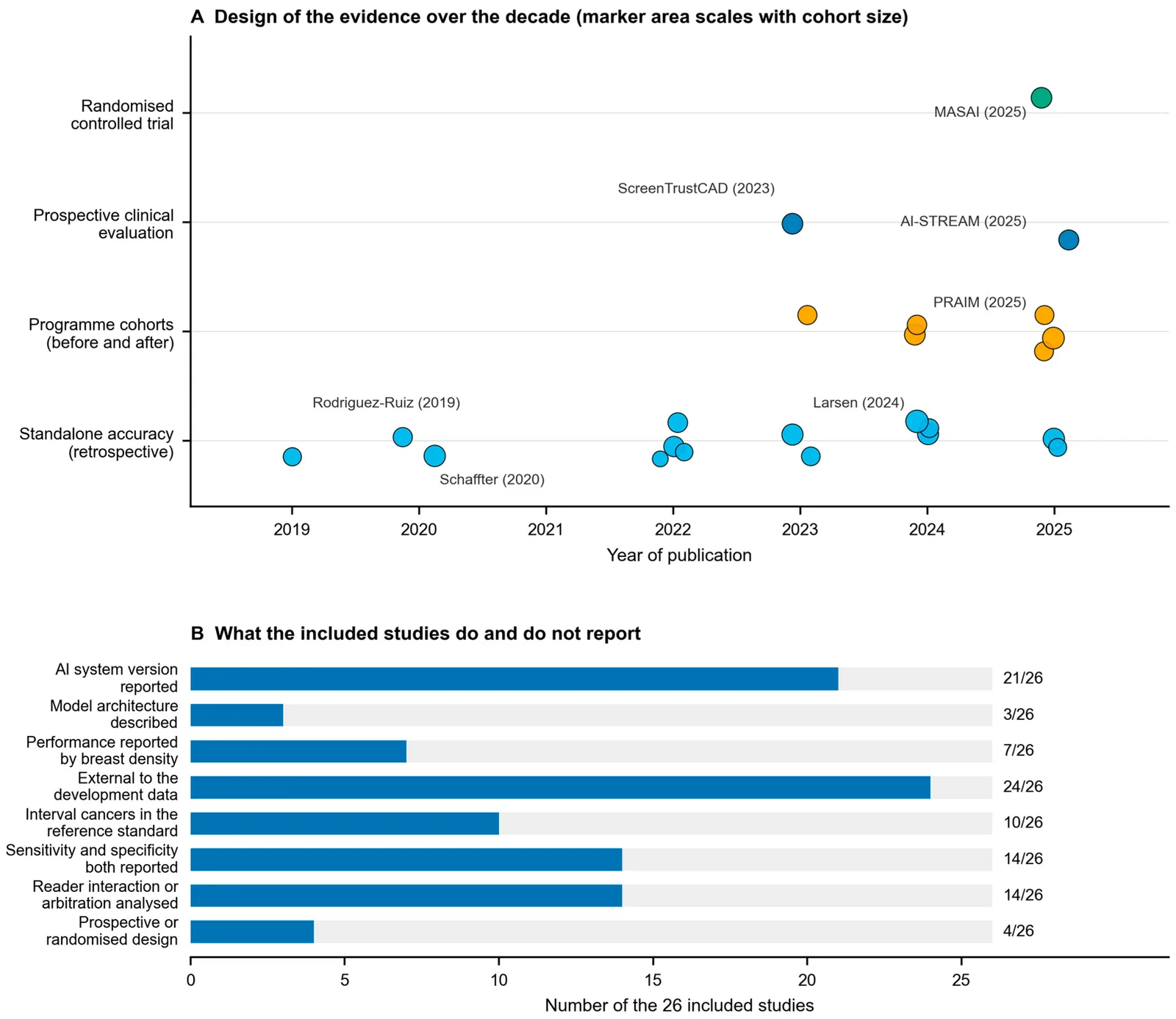
Evidence and technology landscape of the included studies. (**A**) Study design against publication year; marker area scales with the number of examinations. (**B**) Proportion of the 26 included studies reporting each of eight items relevant to appraisal and deployment. Marker colour indicates the design tier: green, randomised controlled trial; dark blue, prospective clinical evaluation; orange, programme cohorts; light blue, retrospective standalone accuracy. Labelled studies: Rodriguez-Ruiz [34], Schaffter [26], ScreenTrustCAD [48], Larsen [43], MASAI [50], AI-STREAM [51] and PRAIM [55].

Figure 2 maps the included studies onto the decade in two panels: the design of the evidence over time, and what the studies do and do not report. Panel B is the more uncomfortable of the two. Twenty-one of 26 studies named the AI system and its version, but only three described the underlying architecture, only seven reported performance separately by breast density, the subgroup in whom mammography performs worst and in whom AI is most often proposed as the remedy, and only four used a prospective or randomised design. The counts behind the panel are published as a study-level table in Data S1 so that each judgement can be checked.

### 3.3. Standalone AI Detection Accuracy (Analysis A)

**Figure 3:**
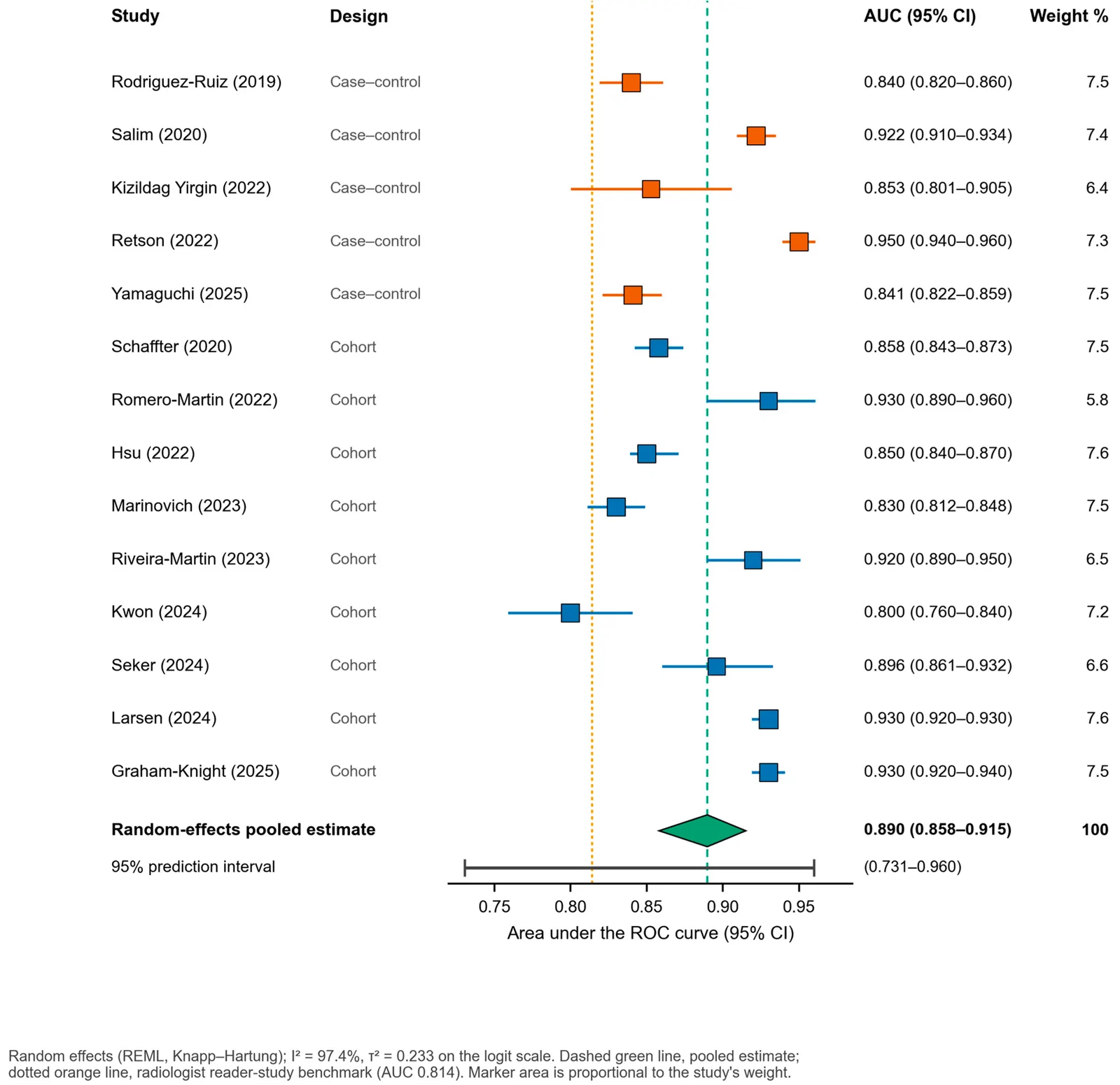
Forest plot of standalone AI diagnostic accuracy. Squares are point estimates, sized in proportion to each study’s weight in the random-effects model; horizontal lines are 95% confidence intervals; the green diamond is the pooled estimate, and its width is the pooled confidence interval. Studies are grouped by design, orange for enriched case–control and blue for consecutive population cohorts. The dashed green line marks the pooled estimate, and the dotted orange line marks the radiologist reader-study benchmark of 0.814. The horizontal bar below the diamond is the 95% prediction interval, which is the range within which the accuracy of a future study would be expected to fall. Weights are given in the right-hand column. Readers who would like a short primer on reading forest plots and on why the prediction interval rather than the diamond answers the question “what should I expect in my programme?” will find one in Lewis and Clarke [65] and in Riley and colleagues [59]; the reporting conventions for diagnostic accuracy syntheses specifically are set out in the PRISMA-DTA elaboration [66]. Data from [25,26,34,35–42,38,43,46,47].

Fourteen studies representing 1,214,885 screening examinations and 9392 cancers reported an AUC that could be pooled. The random-effects estimate was 0.890 (95% CI 0.858–0.915) (Figure 3, Table 2). Individual estimates ranged from 0.800 in a Korean screening cohort [41] to 0.950 in a multicentre multivendor validation of a triage algorithm on enriched data [38].

Between-study heterogeneity was extreme: I^2^ = 97.4% and τ^2^ = 0.233 on the logit scale. The 95% prediction interval, 0.731–0.960, is the number to carry away from this analysis. It says that if the same algorithm class were evaluated in a new screening programme tomorrow, the AUC could plausibly land anywhere between being clearly worse than an average radiologist and near-ceiling. A pooled point estimate of 0.890 conceals that.

The choice of estimator made little difference to the point estimate and a modest difference to its width; all three estimators are reported in Data S1. Leave-one-out analysis moved the pooled estimate within a narrow band, and no single study drove the result. Under the median-system rule, the Swedish case–control study [25] is no longer influential; when the best-performing algorithm is substituted, as in our original submission, the apparent sensitivity of the result to that one study reappears, which is the reason the rule was changed. Omitting that study altogether gives 0.887 (0.852–0.914), and restricting the pool to consecutive population cohorts gives 0.889 (0.846–0.921).

Where the heterogeneity comes from—and does not. Enriched case–control and multi-reader designs (k = 5) gave a pooled AUC of 0.891 (0.831–0.932) and consecutive population cohorts (k = 9) gave 0.889 (0.854–0.916); the difference was negligible (z = 0.08, *p* = 0.94). This was not what we expected, and it is worth stating plainly: in this pool, enriched sampling did not inflate AUC relative to consecutive sampling. The explanation offered in our original submission, that heterogeneity was driven by mixing enriched and cohort designs, is not supported once the pool is large enough to test it.

Grouping by region produced the widest separation of any covariate we examined. The four European cohorts gave 0.928 (0.921–0.933) with I^2^ = 0.0%; the four North American studies gave 0.906 (0.843–0.945) with I^2^ = 98.1%; the six studies from the rest of the world gave 0.840 (0.820–0.857) with I^2^ = 58.7%. We report this as an exploratory observation and would caution against reading it as evidence that accuracy transfers poorly by geography. The European subgroup contains four studies, and region here is entangled with almost everything else that could matter: which programmes had the data infrastructure to run an evaluation, which commercial systems were deployed and at what version, whether reading is single or double, and whether the design was an enriched sample or a consecutive cohort. Three of the four European studies evaluated systems developed on European data, so a vendor-and-setting effect and a regional effect cannot be told apart in these studies. The observation is a hypothesis for a prospective multi-region evaluation to test, which is exactly what Section 4.2 proposes, not a finding about geography.

Meta-regression found no association between publication year and accuracy: β = +0.020 logit units per year (95% CI −0.143 to +0.184, *p* = 0.79), with 0% of between-study variance explained. Adding design as a covariate did not help (β = +0.023, *p* = 0.94), and neither did the two together. Reported standalone accuracy has not measurably improved across 2019–2025. For that reason, the figure plotting AUC against publication year that appeared in our original submission has been removed. It invited a reading of temporal improvement that the data do not support, since the apparent upward drift reflects which populations happened to be studied in which years rather than any change in the technology.

Egger’s regression gave an intercept of −1.72 (t = −0.45, *p* = 0.66), and the funnel plot (Figure S2) is symmetrical. There is no evidence of small-study effects in Analysis A.

For context, the average radiologist AUC in the reader-study setting was 0.814 [34], and the 2023 meta-analysis by Yoon et al. reported a pooled standalone AI AUC of 0.87 against 0.81 for radiologists [20]. Our pooled estimate is close to theirs, which is unsurprising given the partial overlap of included studies; the contribution here is not a different number but a demonstration, on a pool large enough to test it, that the number does not generalise.

**Table 2:** Standalone diagnostic accuracy: AUC for AI in each study, against the comparator reported in that same study.

Study (Year) [Ref]	Design	AI AUC (95% CI)	Comparator in the Same Study	Comparator AUC
Rodriguez-Ruiz (2019) [34]	Enriched case–control	0.840 (0.820–0.860)	Mean of 101 radiologists	0.814
Salim (2020) [25]	Enriched case–control	0.922 (0.910–0.934)	Not reported on the AUC scale	–
Schaffter (2020) [26]	Population cohort	0.858 (0.843–0.873) *	Not reported on the AUC scale	–
Kizildag Yirgin (2022) [35]	Enriched case–control	0.853 (0.801–0.905)	Not reported on the AUC scale	–
Romero-Martin (2022) [36]	Population cohort	0.930 (0.890–0.960)	Not reported on the AUC scale	–
Hsu (2022) [37]	Population cohort	0.850 (0.840–0.870)	Not reported on the AUC scale	–
Marinovich (2023) [39]	Population cohort	0.830 (0.812–0.848) *	Radiologists interpreting the same screens	0.930
Riveira-Martin (2023) [40]	Population cohort	0.920 (0.890–0.950)	Not reported on the AUC scale	–
Kwon (2024) [41]	Population cohort	0.800 (0.760–0.840)	Radiologist BI-RADS assessment	0.740 (0.700–0.780)
Seker (2024) [42]	Population cohort	0.896 (0.861–0.932)	Not reported on the AUC scale	–
Larsen (2024) [43]	Population cohort	0.930 (0.920–0.930)	Not reported on the AUC scale	–
Graham-Knight (2025) [46]	Population cohort	0.930 (0.920–0.940)	Not reported on the AUC scale	–
Yamaguchi (2025) [47]	Enriched case–control	0.841 (0.822–0.859)	Not reported on the AUC scale	–
Retson (2022) [38]	Enriched case–control	0.950 (0.940–0.960)	Not reported on the AUC scale	–
Pooled, random effects	14 studies	0.890 (0.858–0.915)	–	–
95% prediction interval		0.731–0.960		

* Confidence interval approximated from case and control counts by the method of Hanley and McNeil. I^2^ = 97.4%; τ^2^ = 0.233 on the logit scale. The pooled AI AUC is not compared with a radiologist benchmark drawn from a different study; where a within-study comparator exists it is shown on the same row, and it is the only comparison that is internally valid.

### 3.4. Sensitivity and Specificity (Analysis A2)

Nine studies contributed a reconstructable 2 × 2 table, covering 5550 cancers. The bivariate model gave a summary sensitivity of 73.3% (95% CI 64.5–80.6) at a summary specificity of 92.4% (95% CI 86.8–95.8), with a summary diagnostic odds ratio of 33.5 (Table 3, Figure S1). Between-study standard deviations were 0.62 on the logit sensitivity scale and 0.93 on the logit specificity scale, and the correlation between them was −0.75, the expected negative relationship produced by threshold variation.

These figures reframe the AUC result. A specificity above 92% is comfortable, but a summary sensitivity of 73% means that roughly one in four screen-relevant cancers was not flagged by the algorithm at the operating point each study chose. That is broadly comparable to a single human-first reader, and it is well short of what a programme achieves through double reading with arbitration. It is also the reason no included study proposes AI as a sole reader.

The threshold rule differed substantially between studies, and this is a genuine obstacle rather than a technicality. Three studies matched the operating point to a human reader’s specificity, two optimised the Youden index on the same data used for evaluation, which inflates both measures, and four used a vendor-recommended or model-internal cut-off. Table 3 reports the rule alongside each estimate, and the spread of those rules is why the between-study standard deviations are as large as they are. The summary point should be read as the average of nine different questions, not as one question answered nine times.

### 3.5. AI-Integrated Reading Versus Standard Reading (Analysis B)

**Figure 4:**
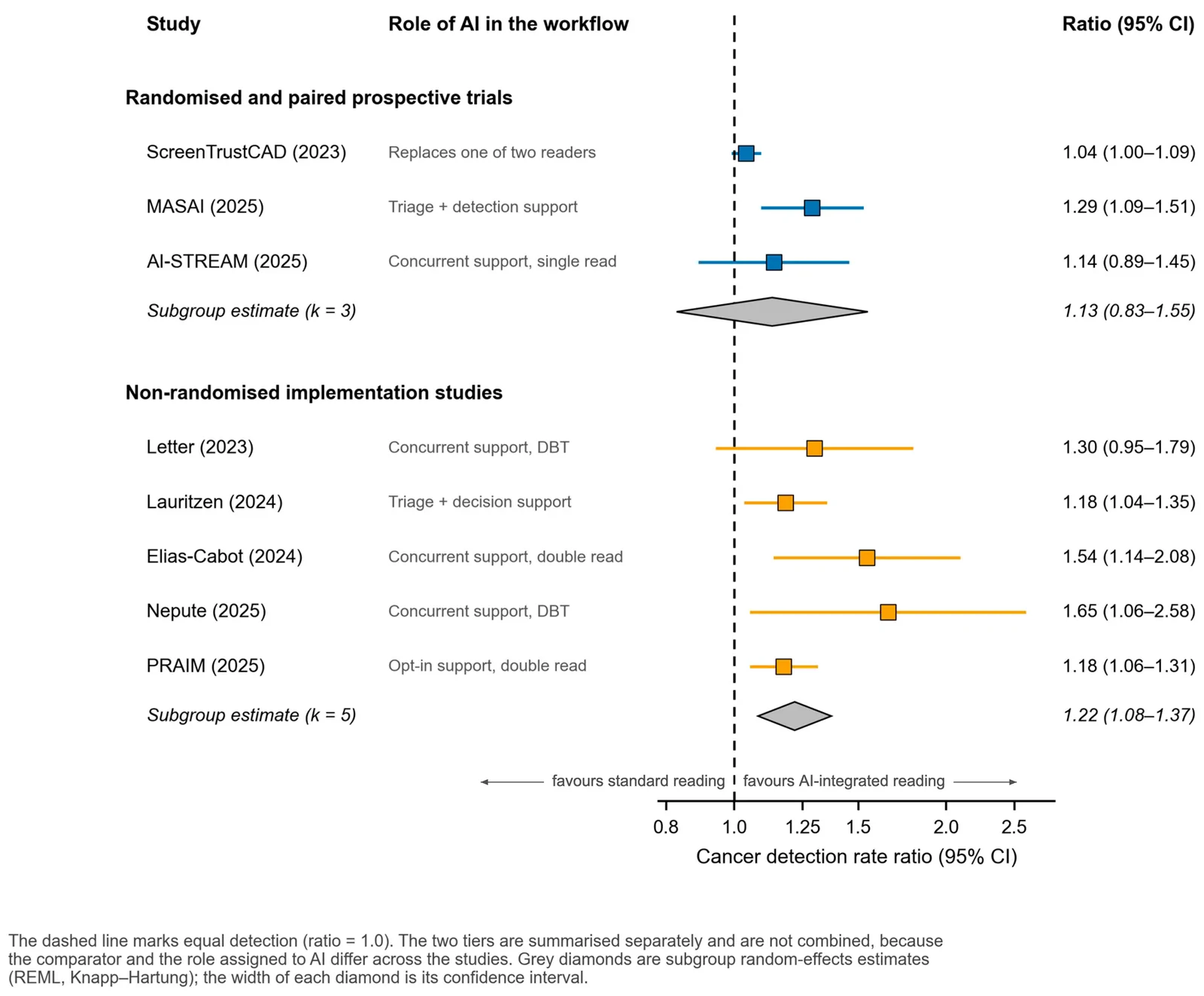
Forest plot of the cancer detection rate ratio for AI-integrated versus standard reading, stratified by evidence tier. The vertical dashed line marks equal detection (ratio 1.0). Grey diamonds are subgroup random-effects estimates and their width is the confidence interval; the two tiers are deliberately not combined into an overall estimate, because the comparator and the role assigned to AI differ across the studies. The role of AI in each reading workflow is stated for every study. Blue squares, randomised and paired prospective trials; orange squares, non-randomised implementation studies; the arrows below the axis indicate the direction favouring standard or AI-integrated reading. Data from [48,50,51,52,53,54–55].

Eight studies compared cancer detection between an AI-integrated reading workflow and standard care (Figure 4, Table 4). They are reported in two tiers, which are not combined.

Randomised and paired prospective trials (k = 3). The MASAI randomised controlled trial randomised 105,915 women in the Swedish national programme to AI-supported screen reading or standard double reading, and reported cancer detection rates of 6.4 versus 5.0 per 1000, a ratio of 1.29 (95% CI 1.09–1.51; *p* = 0.0021), together with a higher positive predictive value of recall (ratio 1.19), no significant increase in false positives and a 44.2% reduction in screen-reading workload [50,62]. The ScreenTrustCAD prospective paired-reader trial found that replacing one of two radiologists with AI was non-inferior for cancer detection (relative proportion 1.04, 95% CI 1.00–1.09), with AI alone also non-inferior (0.98, 0.93–1.04) [48]. The AI-STREAM prospective multicentre cohort, conducted in Korea’s national programme in a single-reading setting, found a 13.8% higher detection rate with AI-CAD support (5.70 versus 5.01 per 1000) and no significant change in recall [51].

Pooled across the three, the detection rate ratio was 1.13 (95% CI 0.83–1.55), with I^2^ = 69.9%. We report that figure for completeness and advise against using it. The three trials asked different questions in different reading systems, and the pooled interval spans both a reduction and a large increase in detection. The randomised comparison is the one that supports a causal claim, and it stands alone.

Non-randomised implementation studies (k = 5). Five studies compared reading with and without AI outside a randomised or paired design. The largest by far is PRAIM, a prospective evaluation across twelve German screening sites, in which 463,094 women were screened and radiologists chose voluntarily whether to use AI support; detection was 6.7 versus 5.7 per 1000, a ratio of 1.18 (1.06–1.31), with recall slightly lower and non-inferior [55]. The others are three before-and-after programme comparisons, in Denmark’s Capital Region [52], a Spanish programme reading both DM and DBT [53] and a US tomosynthesis practice [54], and one concurrent site-controlled comparison of tomosynthesis screening [49]. Pooled, the tier gives 1.22 (1.08–1.37), with I^2^ = 19.0%.

That estimate is both larger and far more precise than the randomised one, and the reader should treat the contrast with suspicion rather than reassurance. Every design in this tier is open to the same family of confounders: secular trends, changes in screening interval, reader learning, and, in PRAIM, self-selection, since the radiologists who opted into AI support may differ systematically from those who did not. Selective publication of successful implementations pushes the same way. Precision is not protection against any of that, and a tight interval around a confounded estimate is still a confounded estimate. GRADE rates this tier very low.

Because the two United States tomosynthesis reports [49,54] share analytic collaborators and may draw on overlapping practices, we repeated the tier estimate omitting each in turn: 1.21 (1.06–1.37) without the first, and 1.21 (1.03–1.42) without the second. Neither omission changes the conclusion.

Does the reading system matter? Reviewer comment prompted us to look at whether the European double-reading context and the single-reading context found in Korea and much of the United States can be treated as one population. They cannot. The three randomised and paired prospective studies assigned AI three different roles (triage plus detection support inside double reading, replacement of one of two readers, and concurrent support in single reading), and the comparator changes accordingly. Figure 4 labels the role for every study. The gains reported in single-reading systems (AI-STREAM, +13.8%) and in double-reading systems with AI triage (MASAI, +29%) are not measuring the same quantity, and a programme considering adoption should look to the study whose reading system matches its own.

### 3.6. Recall, Workload and Other Programme Outcomes (Analysis C)

Six studies reported recall rates for both arms. The pooled recall rate ratio was 0.95 (95% CI 0.81–1.12) with I^2^ = 90.9%, and the direction of effect was not consistent: recall fell in the Danish programme (0.80, 0.74–0.85) and the US tomosynthesis practice (0.79, 0.70–0.89), rose slightly in the Spanish programme (1.13, 1.02–1.26), and was essentially unchanged in MASAI (1.08, 0.99–1.17), PRAIM (0.97, 0.94–1.02) and the site-controlled tomosynthesis comparison (1.00, 0.80–1.30). A large US cohort study makes the same point from the other direction: at either of two operating thresholds, AI achieved a high negative predictive value but recalled more women than the radiologists it was compared with [67]. Whether AI increases or decreases recall appears to depend on where the triage threshold is set and on how discordance is arbitrated, not on the algorithm.

Screen-reading workload reductions were reported as 44.2% in MASAI [50], 62.6% in a Danish simulation [68], 41.4% in the BreastScreen Western Australia validation [39] and 38.3–43.7% across three UK regions in the ARIES study [69]; a meta-analysis of triage deployments of one system reported reductions of a similar order [70]. We did not pool these. The workload saving is set by the operator when the triage threshold is chosen; it is a configuration parameter, not a property of the technology, and a pooled estimate would imply otherwise.

Interval cancers were addressed by 10 of 26 studies. The consistent finding is that AI flags a substantial minority of cancers that human readers missed: 41.2% of interval cancers were flagged by standalone AI in ARIES [69], 44.6% at a 10% triage threshold in BreastScreen Norway [43], and, in the Dutch programme, the interval and future cancers detected only by AI were more often invasive and larger at the time they eventually surfaced [71]. A Swedish case series of 429 interval cancers put a figure on the ceiling: at a threshold corresponding to a 10% recall rate, AI correctly localised lesions on the preceding mammogram in enough cases to reduce the interval-cancer rate by an estimated 19.3% (15.9–23.4), falling to 11.2% at a 4% recall threshold [72]. Whether actioning those flags would convert into a mortality benefit, or mainly into more overdiagnosis and more recalls, is exactly what the randomised trials are still following up.

### 3.7. Risk of Bias and Certainty of Evidence

**Figure 5:**
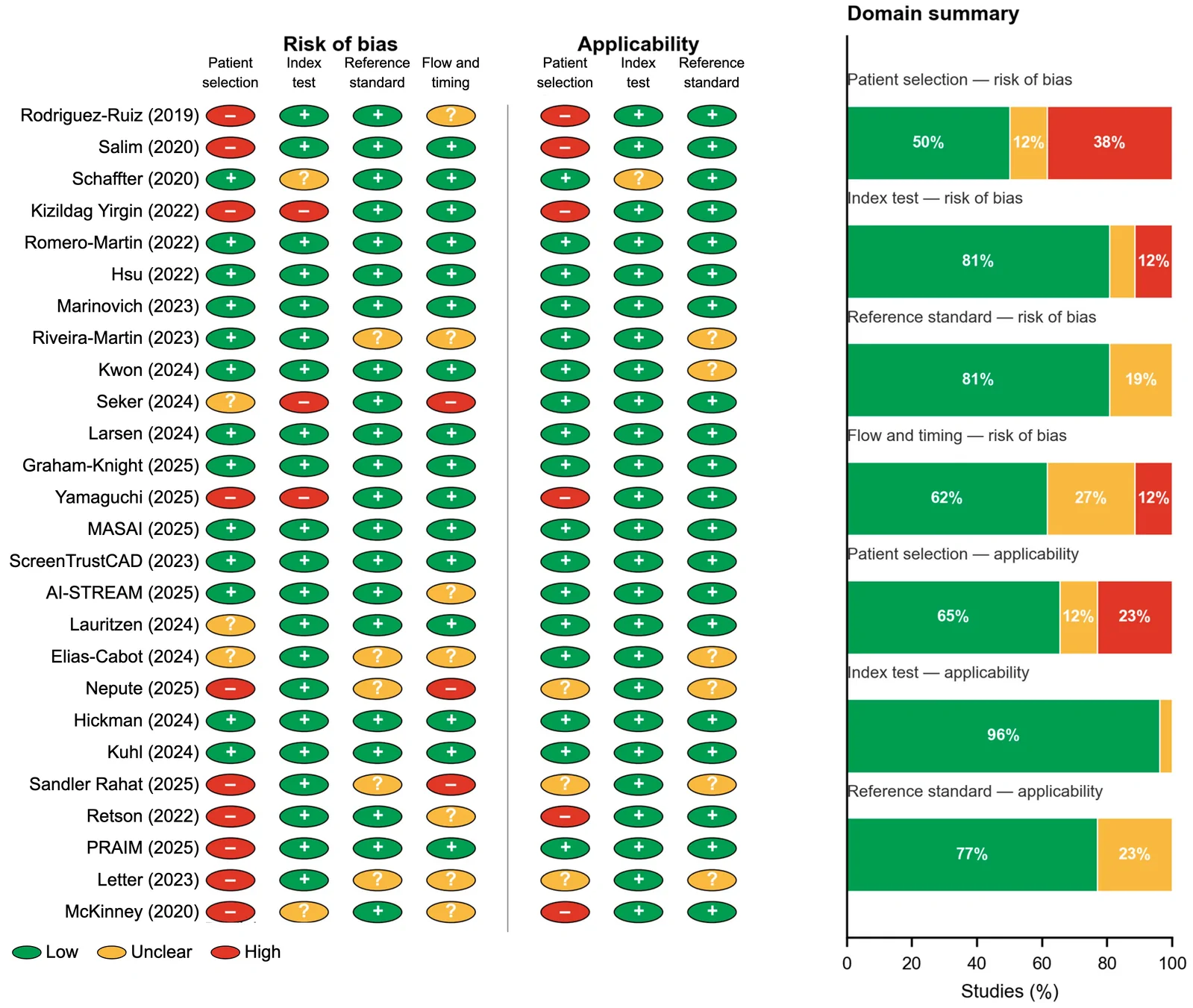
QUADAS-2 assessment. Left, study-level traffic-light plot across the four risk-of-bias and three applicability domains. Right, domain-level summary as a percentage of studies. Green plus, low risk or low concern; amber question mark, unclear; red minus, high risk or high concern. The plot follows the conventional QUADAS-2 layout; readers unfamiliar with it will find the domain definitions and the signalling questions behind each judgement in the original tool description [27] and a practical account of how such summaries are built and read in the documentation of the robvis package [73]. Data from [15,25,26,43–53,45,48,50].

QUADAS-2 judgements for all included studies are shown in Figure 5, and justified study by study in Table S2. Patient selection remained the weakest domain: 10 studies were at high risk, driven by enriched case–control sampling, by non-contiguous observation periods in the before-and-after comparisons, and by the voluntary reader-level uptake of AI in the largest implementation study. Three studies were at high risk in the index-test domain because the decision threshold was optimised on the same data used for evaluation. The reference-standard domain was the strongest, with 81% of studies at low risk, most having ascertained outcomes by registry linkage with follow-up. On applicability, patient selection again raised the most concerns, driven by enriched sampling and by one study that mixed screening with diagnostic examinations.

GRADE certainty was very low for pooled standalone AUC, downgraded for risk of bias, indirectness and very serious inconsistency; very low for summary sensitivity and specificity, downgraded mainly for the incomparability of operating thresholds; low for the pooled prospective detection rate ratio; very low for the non-randomised implementation estimate and for recall; and moderate for the MASAI trial considered on its own, downgraded once for indirectness because a single programme, a single system and a single reading protocol were studied. The complete evidence profile, with the reason recorded for each downgrade, is shown in Table S3.

## 4. Discussion

Over a decade, AI for screening mammography moved from retrospective demonstrations of radiologist-level accuracy to randomised evidence that AI-integrated reading can preserve or increase cancer detection while roughly halving the reading workload. That is a fast trajectory for a screening technology. However, the central finding of this review is not the pace of the field; it is the gap between what the pooled accuracy figure appears to promise and what the evidence will actually support.

Accuracy is high on average and untransferable in particular. The pooled AUC of 0.890 sits comfortably above the reader-study radiologist benchmark of about 0.814. The prediction interval, 0.731–0.960, does not. Two findings sharpen the point. First, publication year explained none of the between-study variance, so the common assertion that AI accuracy has improved year on year is not supported by the studies that have actually reported. Second, the studies cluster by setting in a way that no covariate we could model explains away: four European cohorts agreed with one another almost exactly, while studies from elsewhere pooled some nine AUC points lower. We would not attribute that to geography as such, because region travels with vendor, version, reading protocol and design in these data, and four studies cannot separate them. What it does show is that where a system was developed and tuned is not incidental to how it performs. The UK external validation, in which three commercial algorithms behaved differently on the same cases [44], the Canadian evaluation, which found accuracy falling with increasing breast density [46], and our own single-centre ResNet50 analysis, which reached an AUC of 0.93 in exactly the kind of retrospective setting that flatters an algorithm [13], all point the same way. The practical consequence is that a pooled figure from a review, including this one, is not a substitute for local validation before deployment.

The strongest evidence is at the programme level, and it is thinner than it looks. MASAI, ScreenTrustCAD and AI-STREAM shifted the question from whether AI can read a mammogram to what happens to detection, recall and workload when it is embedded in a real programme [48,50,51,62]. The signal across them is a large, reproducible workload reduction with non-inferior and, in the randomised trial, superior detection, without a false-positive penalty. That is the profile of a second reader or a triage tool. It is not the profile of an unsupervised replacement, and no included study makes that claim. What the programme-level evidence does not yet include is a second randomised trial in a different health system, which is why the certainty for the pooled randomised and paired estimate is low even though the certainty for MASAI alone is moderate.

The non-randomised tier deserves a separate comment, because at first glance it looks like the stronger evidence. It now contains five studies and nearly 700,000 women, it pools to a 22% increase in detection with a tight interval, and its heterogeneity is minimal. None of that makes it more trustworthy than the randomised comparison. The largest contributor, a nationwide German implementation of 463,094 women, allocated AI support by letting radiologists opt in [55]; readers who chose to use it may differ in experience, workload or caseload from those who did not, and no design feature of the study can rule that out. The other four are before-and-after or site-controlled comparisons in which secular trends and reader learning run alongside the intervention. When a body of non-randomised evidence is both larger and more optimistic than the randomised evidence it sits beside, the most economical explanation is usually the one about confounding, and we read it that way.

Replacing readers is not the same as improving care. A reviewer put this well: a technology that substitutes for a human does not automatically improve medicine. Two observations from this review support the caution. Recall moved in both directions across programmes, which means the same technology can either reduce or increase the harms of false positives depending on how it is configured. And outcomes depend heavily on how radiologist–AI discordance is arbitrated: an experimental study showed that incorrect AI BI-RADS suggestions degrade reader performance through automation bias [74], simulated-arbitration analyses show that the discordance rule materially changes programme-level accuracy [75], and a reader study of wide-angle tomosynthesis found that decision support raised reader AUC only when the support was accurate [76]. A population-wide Danish simulation makes the same point about placement rather than capability: replacing the first reader, the second reader or both with the same algorithm produced materially different sensitivity, arbitration and workload, so where AI sits in the workflow changes the outcome as much as how well it reads [77]. The most defensible reading of the evidence is that AI adds most value where it complements human reading, by flagging cancers humans miss and removing normal examinations from the queue, rather than where it substitutes for it.

Calibration, vendors and versions. Most included studies used a commercial system, but only three reported the model architecture and several did not report the version. Systems are updated between publications, and an evaluation of Transpara 1.7.0 does not licence a claim about Transpara generally. Two studies that examined this directly found that AI risk scores are not calibrated identically across acquisition vendors [40], and a study of density classification found that performance degraded markedly when a model trained on one manufacturer’s images was applied to another’s [78]. A UK regional programme made the practical consequence unusually concrete: applying a vendor’s pre-specified threshold produced a recall rate of 48.3%, which fell to 13.0% after local calibration, and rose roughly threefold again after a software upgrade on the mammography equipment, so that a separate threshold was needed per software version [79]. A programme buying an AI system is buying a specific version, calibrated for specific hardware, and neither the version nor the calibration is stable over the life of a screening contract.

Equity and external validation across populations. Most evidence still comes from European and North American programmes. The Japanese, Korean, Turkish and Australian studies broaden the base, but three of those four were retrospective, and none of the included studies reported performance by ethnicity as a pre-specified subgroup. The single study that stratified by ethnicity found the same performance disparities in the AI workflow as in human double reading, which is reassuring about relative harm and not at all reassuring about absolute equity [69]. Bias in these models can persist through naive mitigation and can even be introduced by it [80], and work in adjacent imaging domains shows how difficult it is to correct once present [81]. Prospective subgroup evaluation remains the missing piece. Related to this is spectrum bias. Five of the studies pooled here used enriched case–control sampling, in which cancers are over-represented relative to a screening population. The conventional expectation is that this inflates AUC. In our pool it did not, which we take as a caution against treating design labels as a substitute for looking at the data, but the applicability concern stands regardless: an algorithm evaluated on a case mix that no programme encounters has not been evaluated in the setting where it will be used.

Explainability, accountability and regulation. Almost none of the included studies reported an explainability component that was evaluated as part of the accuracy claim, and a scoping review of explainable AI in breast imaging reached the same conclusion for the wider literature: saliency and attention methods are commonly displayed but rarely validated against what a radiologist needs to make a decision [82]. This matters for accountability. If a programme replaces a human reader with an algorithm, the question of who is answerable for a missed cancer does not disappear; it moves. Regulatory frameworks are catching up unevenly. A recent evidence-based review of FDA-cleared mammography AI devices found that clearance rests predominantly on retrospective reader studies rather than on prospective clinical outcomes [83], and, in Europe, the Medical Device Regulation and the AI Act now place these systems in a high-risk class with post-market surveillance obligations that most published evaluations were not designed to satisfy [84,85]. Health-technology assessment frameworks are only beginning to be applied to this technology at all [86].

Health economics. Decision-analytic modelling of the UK programme suggests that risk-stratified, AI-guided screening could be cost-saving while adding quality-adjusted life-years [87]. That analysis is a model, not a trial, and its inputs are drawn from the same accuracy literature whose dispersion this review documents. Cost-effectiveness conclusions inherit the uncertainty of their accuracy inputs, and a prediction interval spanning 0.73 to 0.96 in AUC is a wide input.

Beyond detection. AI also extends past the reading task. Deep-learning risk models such as Mirai discriminate future cancer better than questionnaire models and have been validated across institutions [88,89]; multimodal models combine imaging with clinical data [90]; and the ScreenTrustMRI randomised trial used an AI score to triage the highest-risk 6.9% of women with negative mammograms to supplemental MRI, detecting roughly four times more cancers per 1000 MRI examinations than a density-based strategy [91]. On the modality side, tomosynthesis is becoming the screening standard [92,93] and contrast-enhanced mammography is expanding [94]; where AI fits into each workflow is still being worked out [95].

What a multi-region trial would have to look like. A reviewer asked what we would actually recommend for a multicentre trial across regions, and the subgroup pattern in Section 3.3 is the reason the question matters: the only honest way to separate a regional effect from a vendor, version, protocol and case-mix effect is to hold some of them fixed by design. We would propose a cluster-randomised trial with screening units as the unit of randomisation, stratified by country and by baseline reading protocol, and powered on interval-cancer rate rather than on cancer detection rate, since detection is an intermediate outcome and the trials that have reported already agree on it. Three design features would do most of the work. The same AI system, at a version frozen for the trial and with the version recorded, should be deployed in every region, so that regional comparisons are not confounded by product. The operating threshold should be set once, centrally, by a rule stated in the protocol rather than tuned locally, with any local recalibration recorded as a protocol deviation and analysed as such. And the arbitration rule for radiologist–AI discordance should be identical across sites, because Section 3.6 shows that this rule, not the algorithm, drives the recall outcome. Sites should span at least the double-reading and single-reading traditions and should be selected to give a pre-specified minimum representation by breast density and by ethnicity, with subgroup analyses declared in advance rather than assembled afterwards. A registry linkage of at least two years is needed for interval cancers, and the trial should publish its full extraction and analysis code, because the reproducibility gap documented in this review is itself an obstacle to synthesis. A trial built this way would answer the transferability question that none of the studies reviewed here can answer.

Validating a moving target. The same reviewer raised the harder problem: how would one control validation when the technology itself keeps changing? A model that has been revalidated once is not validated for the life of a contract, because three things move underneath it. The system changes when the vendor ships a new version. The input changes when a site replaces or upgrades its mammography equipment, which is exactly the effect that tripled recall in the UK programme described above [79]. And the population changes as screening intervals, invitation policy and case-mix drift. This is the general problem of dataset shift, which has a growing literature in clinical machine learning [96,97] and an emerging regulatory vocabulary in the lifecycle approach to software as a medical device [98].

With unlimited resources, we would treat validation as a standing programme function rather than a one-off study, built on four elements. A frozen local reference set, assembled once from the programme’s own consecutive screening examinations with registry-confirmed outcomes, against which any new version is scored before deployment, and which is refreshed on a stated schedule. Continuous monitoring of the score distribution itself, since a shift in the distribution of AI scores is detectable within weeks and does not require waiting for cancer outcomes, whereas a shift in detection rate does. Pre-agreed control limits on recall rate, detection rate and arbitration rate, with a documented response when a limit is breached, in the way that programmes already monitor reader performance. And a change-control rule that treats a vendor version upgrade or an equipment change as an event requiring re-validation against the frozen reference set, not as routine maintenance. None of this is exotic; it is the quality-assurance apparatus that organised screening already applies to human readers and to image quality, extended to a component that happens to be software. What is missing is not the method but the expectation that it will be applied, and the reporting standard that would let one programme learn from another’s monitoring data.

### 4.1. Limitations

This review has several limitations, and two of them are consequential.

The first is the search. Embase, Scopus, Web of Science, IEEE Xplore and the Cochrane Library were not searched, because they are not available to us and cannot be queried reproducibly. Adding PubMed to Europe PMC, trial registries and citation searching recovers much of what those databases would return for a clinically framed question, and the citation search of 750 records yielded two additional eligible studies, which suggests the retrieved pool is close to saturated. It does not prove it. Studies indexed only in engineering databases are unlikely to meet our eligibility criteria, which require a screening population and a follow-up-based reference standard, but we cannot demonstrate that no eligible study was missed.

The second is the screening process, and the second round of review made it possible to measure the problem rather than only describe it. The first pass over 1743 records was executed by an explicit rule set rather than by two independent human raters. Verifying it against an independently specified second procedure (Section 2.4) returned a Cohen’s κ of 0.47, which is moderate agreement at best, and adjudication of the 250 discordant records recovered three eligible studies the first pass had missed. Two of those three are now among the more consequential studies in the review. Counting the studies that entered by other routes, the first pass identified 18 of the 24 eligible studies that were present in the record set, a sensitivity of 75%.

We report that plainly because it bears on how the review should be read. Screening built on a single deterministic rule set is auditable and exactly reproducible, and every exclusion is published with the rule that produced it, but it is not equivalent to duplicate independent screening, and it demonstrably missed eligible work. The verification exercise raises our confidence that the current pool is close to complete, since a deliberately more inclusive second procedure applied to the whole record set found three additions and nothing else, but it cannot establish that no eligible study remains outside it. For the full-text stage, the duplicate judgements were reconciled to consensus as the assessment proceeded rather than recorded separately first, so no agreement statistic can be computed for that stage after the fact.

Beyond those: non-English publications were excluded for practical reasons. Extreme heterogeneity means the pooled accuracy estimate should be read as descriptive rather than as a performance expectation. A hierarchical summary ROC curve could not be fitted because every study reports a single, differently chosen threshold. Analyses B and C rest on small numbers of studies in each stratum and could not be tested for small-study effects. Five of the eight implementation studies are non-randomised, and in these secular trends, reader learning and, in the largest of them, voluntary uptake of AI cannot be separated from the effect of the technology. Only one randomised trial has reported, from a single country with a single AI system. The regional pattern in Analysis A is exploratory and confounded by vendor, version, reading protocol and design, and should not be read as a finding about geography. And the endpoints that ultimately matter, namely interval-cancer rate, mortality and overdiagnosis, remain in follow-up in every trial included here.

### 4.2. Implications and Future Research

For practice, the evidence supports the controlled implementation of AI as a triage or second-reader tool within organised programmes, on three conditions: local validation on the programme’s own population and hardware before go-live, prospective monitoring of detection and recall against pre-agreed thresholds, and an explicit rule for arbitrating radiologist–AI discordance. Programmes should expect to re-validate when the vendor ships a new version.

For research, the priorities follow directly from what this review could not resolve. Randomised evidence is needed from a second health system and a second AI system, and the design we would argue for is set out above. Our registry search identified six evaluations that had enrolled but not yet reported (Table S5), among them randomised trials in Norway (150,000 women), the United States (400,000) and Czechia (8000), and two completed European programme evaluations awaiting publication. Between them, these will roughly quadruple the randomised evidence base within a few years. Long-term follow-up of the reporting trials for interval cancer and mortality is the single most valuable output the field can produce in the next five years. Prospective evaluation of performance by breast density and by ethnicity should be pre-specified rather than exploratory. Reporting should follow TRIPOD+AI and CLAIM, and should state the system version, the architecture, the threshold-selection rule and the arbitration protocol as a minimum. Programmes deploying these systems should publish their monitoring data, including score distributions and control-limit breaches, because that is the evidence base for validating a moving target and at present it does not exist. Finally, the field would benefit from agreeing on sensitivity and specificity at a stated operating point as the primary reported measures, with AUC secondary; the inability to fit a hierarchical model to seven years of published work is a reporting failure, not a statistical one.

## 5. Conclusions

Between 2015 and 2025, AI for screening mammography matured from retrospective parity with radiologists to randomised evidence of substantial workload reduction with maintained or increased cancer detection. Pooled standalone accuracy is high, at an AUC of approximately 0.89, but its 95% prediction interval spans 0.73 to 0.96, and neither publication year nor study design explains that dispersion, so the pooled figure cannot be transferred to a new programme without local validation. Increased cancer detection has been demonstrated in one randomised trial, in one country, with one AI system; the before-and-after programme audits report larger gains that should be treated with corresponding caution. Interval-cancer and mortality outcomes are still awaited in every trial.

Taken together, the weight of a decade of evidence positions AI as a well-supported second-reader and workload-reduction tool for organised screening programmes that validate it locally and monitor it prospectively. It does not position AI as an autonomous replacement for the radiologist, and the evidence needed to support that claim has not been produced. Priorities for the next decade are randomised replication in a second health system, long-term follow-up for interval cancer and mortality, prospective evaluation across breast density and ethnicity, standardised reporting under frameworks such as TRIPOD+AI, and health-economic and equity evaluation of risk-stratified, AI-guided screening.

## Figures and Tables

**Table 1:** Characteristics of the 26 included studies and their contribution to each synthesis.

Study (Year) [Ref]	Country	Design	Examinations	Cancers	AI System	Comparator	Analyses
Rodriguez-Ruiz (2019) [34]	Seven countries	MRMC reader study, enriched	2652	653	Transpara	101 radiologists	A
Salim (2020) [25]	Sweden	Retrospective case–control	8805	739	AI-2 (median of three vendors)	First and second reader	A, A2
Schaffter (2020) [26]	USA	Retrospective cohort (DREAM)	144,231	952	Top-ranked DREAM algorithm	Radiologists	A
McKinney (2020) [15]	UK, USA	Retrospective validation and reader study	28,953	Not reported	Google Health	Radiologists	—
Kizildag Yirgin (2022) [35]	Türkiye	Retrospective case–control	211	110	Commercial AI system	Two radiologists	A, A2
Romero-Martin (2022) [36]	Spain	Retrospective consecutive cohort	15,999	113	Transpara	Single and double reading	A
Hsu (2022) [37]	USA	Retrospective external validation	37,317	Not reported	DREAM challenge ensemble model	Radiologists	A
Retson (2022) [38]	USA	Multicentre multivendor case–control	1255	400	Commercial triage algorithm (cmTriage)	BCSC reader benchmark	A, A2
Marinovich (2023) [39]	Australia	Retrospective population cohort	108,970	760	Commercial AI algorithm	Radiologists	A, A2
Riveira-Martin (2023) [40]	Spain	Retrospective population cohort	Not reported	Not reported	Commercial AI system	Programme readers	A
Kwon (2024) [41]	South Korea	Retrospective population cohort	89,855	143	Lunit INSIGHT MMG	Radiologist BI-RADS	A, A2
Seker (2024) [42]	Türkiye	Retrospective programme cohort	5136	105	Commercial AI system	Two-reader programme	A, A2
Larsen (2024) [43]	Norway	Retrospective national cohort	661,695	4917	Transpara	Programme readers	A
Hickman (2024) [44]	UK	Retrospective screening cohort	26,722	760	Three commercial algorithms	Single and double reading	A2
Kuhl (2024) [45]	Denmark	Retrospective regional cohort	249,402	2033	Commercial AI system	First readers	A2
Graham-Knight (2025) [46]	Canada	Retrospective provincial cohort	136,700	Not reported	Commercial AI algorithm	Radiologists	A
Yamaguchi (2025) [47]	Japan	Retrospective multi-institutional	2059	500	DLADS (SE-ResNet)	Pre-specified 80% target	A, A2
ScreenTrustCAD (2023) [48]	Sweden	Prospective paired-reader trial	55,581	261	Lunit INSIGHT MMG	Two radiologists	B
Letter (2023) [49]	USA	Concurrent site-controlled comparison	Not reported	Not reported	iCAD ProFound AI v2.0	Sites without AI	B, C
MASAI (2025) [50]	Sweden	Randomised controlled trial	105,915	338	Transpara 1.7.0	Standard double reading	B, C
AI-STREAM (2025) [51]	South Korea	Prospective multicentre cohort	24,543	140	AI-CAD	Single reading, no AI	B
Lauritzen (2024) [52]	Denmark	Before-and-after cohorts	118,997	480	Commercial AI system	Double reading before AI	B, C
Elias-Cabot (2024) [53]	Spain	Before-and-after cohorts	23,996	108	Commercial AI system	Double reading before AI	B, C
Nepute (2025) [54]	USA	Before-and-after DBT interpretations	16,729	39	Deep-learning AI support	Conventional CAD	B, C
PRAIM (2025) [55]	Germany	Prospective real-world implementation	463,094	1747	Commercial AI system	Standard double reading	B, C
Sandler Rahat (2025) [56]	Israel	Retrospective audit before and after AI	31,176	Not reported	iCAD version 2.0	Screening before AI	—

A, standalone accuracy (pooled AUC); A2, bivariate sensitivity and specificity; B, cancer detection rate ratio; C, recall rate ratio; em dash, narrative synthesis only. MRMC, multi-reader multi-case; DBT, digital breast tomosynthesis; BCSC, Breast Cancer Surveillance Consortium. MASAI is reported in two publications of the same trial and is counted once. Examinations are screening examinations analysed; for the implementation studies both arms are summed.

**Table 3:** Sensitivity and specificity at the operating point each study selected.

Study (Year) [Ref]	Rule Used to Set the Operating Point	Sensitivity %	Specificity %
Salim (2020) [25]	Cut point set at mean first-reader specificity (96.6%)	67.0	96.6
Kizildag Yirgin (2022) [35]	Youden-optimised risk-score cut-off (34.5%)	72.8	88.3
Marinovich (2023) [39]	Prospective vendor-recommended threshold	67.0	81.0
Kwon (2024) [41]	Probability-of-malignancy cut-off 10%	67.1	93.0
Seker (2024) [42]	Youden-optimised cut-off (30.44)	72.4	92.9
Hickman (2024) [44]	Preset specificity matched to a single human reader	58.9	97.9
Yamaguchi (2025) [47]	Heatmap concentration-gradient cut-off 15%	83.5	84.7
Kuhl (2024) [45]	Cut point matched to mean first-reader specificity	62.6	97.7
Retson (2022) [38]	Vendor default operating point	93.0	76.3
Bivariate summary, 9 studies		73.3 (64.5–80.6)	92.4 (86.8–95.8)

Correlation between logit sensitivity and logit specificity is −0.745; summary diagnostic odds ratio is 33.5. The threshold rules are not equivalent, and the two studies that optimised the Youden index on their own evaluation data will have overstated both measures.

**Table 4:** Programme-level outcomes for AI-integrated versus standard reading.

Study (Year) [Ref]	Tier	Role of AI	CDR with AI	CDR Standard	Detection Rate Ratio (95% CI)	Recall Ratio (95% CI)
MASAI (2025) [50]	Randomised/paired	AI triage plus detection support	6.40	5.00	1.29 (1.09–1.51)	1.08 (0.99–1.17)
ScreenTrustCAD (2023) [48]	Randomised/paired	AI replacing one of two readers	4.70	4.50	1.04 (1.00–1.09)	Not reported
AI-STREAM (2025) [51]	Randomised/paired	AI-CAD concurrent support in single reading	5.70	5.01	1.14 (0.89–1.45)	Not reported
Randomised/paired subgroup					1.13 (0.83–1.55)	
Lauritzen (2024) [52]	Non-randomised	AI triage plus decision support	8.24	6.96	1.18 (1.04–1.35)	0.80 (0.74–0.85)
Elias-Cabot (2024) [53]	Non-randomised	AI concurrent support for double reading	9.00	5.80	1.54 (1.14–2.08)	1.13 (1.02–1.26)
Nepute (2025) [54]	Non-randomised	AI concurrent support for DBT reading	6.10	3.70	1.65 (1.06–2.58)	0.79 (0.70–0.89)
PRAIM (2025) [55]	Non-randomised	AI-supported double reading, radiologist opts in	6.70	5.70	1.18 (1.06–1.31)	0.97 (0.94–1.02)
Letter (2023) [49]	Non-randomised	AI concurrent support for DBT reading	7.30	5.90	1.30 (0.95–1.79)	1.00 (0.80–1.30)
Non-randomised subgroup					1.22 (1.08–1.37)	
Recall, all 6 reporting studies						0.95 (0.81–1.12)

CDR, cancer detection rate per 1000 screening examinations. The two tiers are summarised separately and are not combined. Where a report gives an interval that accounts for the study design, that interval is used; otherwise, intervals are derived from independent Poisson variances and are therefore conservative. Prospective tier I^2^ = 69.9%; non-randomised tier I^2^ = 19.0%; recall I^2^ = 90.9%.

## Data Availability

All data underlying this review are published with the article. The extraction tables, the screening and deduplication logs, the record-level screening adjudication, the reasons for every full-text exclusion, the QUADAS-2 judgements and the analysis and figure scripts are provided as Data S1. Running meta_analysis.py, screening_verification.py, make_adjudication.py, make_fulltext_exclusions.py, make_tables.py and make_figures.py in that order regenerates every estimate, table and figure reported here, including the counts printed in the PRISMA diagram. The protocol and blank extraction form are deposited on protocols.io (https://dx.doi.org/10.17504/protocols.io.q26g7q469lwz/v1).

## Supplementary Materials

The following supporting information can be downloaded at: https://www.mdpi.com/article/10.3390/diagnostics16183045/s1, Table S1, full electronic search strategies for every source with line-by-line yields (PRISMA-S); Section S1, the title-and-abstract screening rule set, the screening log, the independent verification of that stage, the complete 250-record adjudication and the reasons for every full-text exclusion; Table S2, QUADAS-2 judgements and written rationale for all 26 studies; Table S3, GRADE evidence profile for every synthesis outcome; Table S4a–d, the complete extraction tables underlying Analyses A, A2, B and C and the reporting-completeness table behind Figure 2; Table S5, ongoing and unpublished trials identified in ClinicalTrials.gov; Table S6, completed PRISMA 2020 checklist; Table S7, completed PRISMA-DTA checklist; Table S8, blank data-extraction template; Figure S1, summary ROC space for the bivariate model; Figure S2, funnel plot for Analysis A; Protocol S1, pre-registered protocol and extraction form deposited on protocols.io (DOI: https://dx.doi.org/10.17504/protocols.io.q26g7q469lwz/v1); Data S1, screening and deduplication logs, the record-level adjudication and full-text exclusion listings, extraction tables and the analysis code in Python v3.10.

## Abbreviations

AI	Artificial intelligence
AI-STREAM	Artificial Intelligence for Breast Cancer Screening in Mammography
AUC	Area under the receiver operating characteristic curve
BI-RADS	Breast Imaging-Reporting and Data System
CAD	Computer-aided detection
CDR	Cancer detection rate
CI	Confidence interval
DBT	Digital breast tomosynthesis
DLADS	Deep-learning-based automated diagnostic system
DM	Digital mammography
DREAM	Dialogue for Reverse Engineering Assessments and Methods
GRADE	Grading of Recommendations Assessment, Development and Evaluation
HSROC	Hierarchical summary receiver operating characteristic
MASAI	Mammography Screening with Artificial Intelligence
MDR	Medical Device Regulation
MRMC	Multi-reader multi-case
MRI	Magnetic resonance imaging
PIRD	Population, Index test, Reference standard, Diagnosis
PRISMA	Preferred Reporting Items for Systematic Reviews and Meta-Analyses
PRISMA-DTA	PRISMA for Diagnostic Test Accuracy
PRISMA-S	PRISMA Search Reporting Extension
PROBAST+AI	Prediction model Risk-Of-Bias Assessment Tool for artificial intelligence
QUADAS-2	Quality Assessment of Diagnostic Accuracy Studies, version 2
QUADAS-C	QUADAS for comparative accuracy
REML	Restricted maximum likelihood
ROC	Receiver operating characteristic
TRIPOD + AI	Transparent Reporting of a multivariable prediction model for Individual Prognosis Or Diagnosis, AI extension
XAI	Explainable artificial intelligence
