# Supplementary material for: Ten Years of Artificial Intelligence in Screening Mammography: A Systematic Review and Meta-Analysis of Diagnostic Accuracy and Clinical Implementation (Literature Published 2015–2025)
Source: Diagnostics (Basel). 2026 Sep 20;16(18):3045. doi: 10.3390/diagnostics16183045 (PMC13605948; doi:10.3390/diagnostics16183045)
Supplement: Supplementary file 1 [file diagnostics-16-03045-s001.zip › Supplementary_Materials/00_CONTENTS.pdf]

Supplementary Materials — contents

Manuscript: diagnostics-4463959 (third revision) *Ten Years of Artificial Intelligence in Screening Mammography: A Systematic Review and Meta-Analysis of Diagnostic Accuracy and Clinical Implementation (Literature Published 2015–2025)*

Ciurescu S, Buciu V, Ilaș D-G, Pârvănescu R, Șerban D.

New or substantially changed at this revision

Item	Change
Section S1.5	Rewritten. The 133 reports excluded at full-text assessment are now listed report by report in Data S1, with the eligibility criterion each fails, and the tally printed in Figure 1 is generated from that listing
Section S1.6	Rewritten. The adjudication of the 250 discordant screening records is described as the two-stage process it was, and the earlier claim that all 250 were adjudicated by two reviewers is withdrawn. The sensitivity of the first screening pass is corrected from 17 of 20 (about 85%) to 18 of 24 (75%), with the denominator defined
Table S6	Items 8, 16a and 16b updated to reflect the completed adjudication, the balanced flow diagram and the record-level exclusion listing
Data S1	Adds `screening_adjudication.csv` (all 250 discordant records) and `fulltext_exclusions.csv` (all 133 full-text exclusions), together with the two scripts that generate them. The scripts now resolve the data folder whether it is named `data_S1` or `Data_S1`, so they run on a case-sensitive filesystem

Nothing in the analyses changed at this revision. Tables S2, S3, S4a–S4d, S5, S7 and S8, Figures S1 and S2 and Protocol S1 are unchanged from the second revision.

Tables/

File	Cited as	Content
`Table_S1_search_strategies.docx`	Table S1	Line-by-line search strategy and yield for PubMed, Europe PMC, ClinicalTrials.gov and citation searching (PRISMA-S)
`Section_S1_screening_rules.docx`	Section S1	The screening rule set, the screening log, the independent verification of that stage, the complete 250-record adjudication and

		the reasons for every full-text exclusion
`Table_S2_QUADAS2.docx`	Table S2	QUADAS-2 judgements and written rationale for all 26 studies
`Table_S3_GRADE_DTA.docx`	Table S3	GRADE evidence profile for all six synthesis outcomes
`Table_S4_extraction_tables.docx`	Tables S4a–S4d	The complete extraction tables and the reporting-completeness table
`Table_S5_ongoing_trials.docx`	Table S5	Registered evaluations, including six that had not reported
`Table_S6_PRISMA_2020_checklist.docx`	Table S6	Completed PRISMA 2020 checklist
`Table_S7_PRISMA_DTA_checklist.docx`	Table S7	Completed PRISMA-DTA checklist
`Table_S8_extraction_template.docx`	Table S8	Blank data-extraction form

Figures/

File	Cited as	Content
`figureS1_sroc`	Figure S1	Summary ROC space for the bivariate model (k = 9)
`figureS2_funnel`	Figure S2	Funnel plot for Analysis A (k = 14); Egger p = 0.66

PDF is vector; PNG is 400 dpi.

Protocol/

`Protocol_S1.docx` — review protocol and analysis plan, deposited on protocols.io (https://dx.doi.org/10.17504/protocols.io.q26g7q469lwz/v1), with all post-registration amendments listed and dated by review round.

Data_S1/

Everything needed to reproduce the review. `DATA_S1_README.md` inside the folder is the index and states which scripts produced the reported values. In short:

Analysis code. `meta_analysis.py` (authoritative), `screening_verification.py`, `make_adjudication.py`, `make_fulltext_exclusions.py`, `make_tables.py`, `make_figures.py`, and `meta_analysis.R` (a reference implementation using metafor and mada, supplied at a reviewer's request and clearly labelled as not executed by the authors, because R was unavailable in our environment).

Extraction tables. The four CSVs the analyses read, one row per study, each carrying its PubMed identifier and DOI.

Screening records. All 1,743 deduplicated records; the first-pass decision and rule for each; the 250 records on which the two screening procedures disagreed; the final decision, criterion and adjudicating stage for

every one of those 250; and the eligibility criterion behind each of the 133 reports excluded at full-text assessment.

Outputs. The full rendered analysis and the machine-readable summaries that the table and figure scripts read, which is why no number can drift between the analysis, the tables, the diagram and the text.

Run `meta_analysis.py`, then `screening_verification.py`, `make_adjudication.py`, `make_fulltext_exclusions.py`, `make_tables.py` and `make_figures.py`, in that order.
