# Supplementary material for: Ten Years of Artificial Intelligence in Screening Mammography: A Systematic Review and Meta-Analysis of Diagnostic Accuracy and Clinical Implementation (Literature Published 2015–2025)
Source: Diagnostics (Basel). 2026 Sep 20;16(18):3045. doi: 10.3390/diagnostics16183045 (PMC13605948; doi:10.3390/diagnostics16183045)
Supplement: Supplementary file 1 [file diagnostics-16-03045-s001.zip › Supplementary_Materials/Figures/figureS1_sroc.pdf]

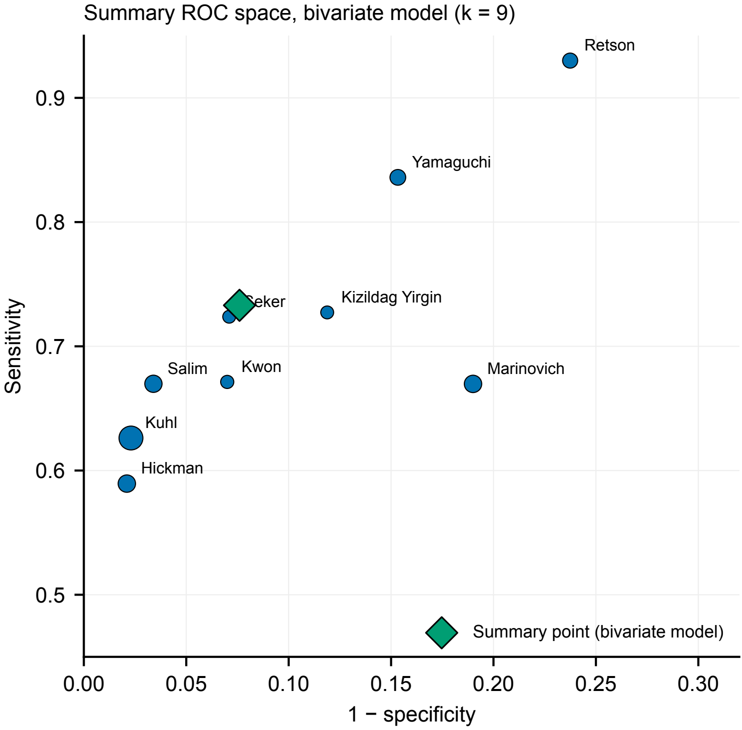
Summary ROC space, bivariate model (k = 9)
Sensitivity
1 − specificity
0.5
0.6
0.7
0.8
0.9
0.00
0.05
0.10
0.15
0.20
0.25
0.30
Retson
Yamaguchi
Ceker
Kizildag Yirgin
Salim
Kwon
Marinovich
Kuhl
Hickman
Summary point (bivariate model)
