# Supplementary material for: Ten Years of Artificial Intelligence in Screening Mammography: A Systematic Review and Meta-Analysis of Diagnostic Accuracy and Clinical Implementation (Literature Published 2015–2025)
Source: Diagnostics (Basel). 2026 Sep 20;16(18):3045. doi: 10.3390/diagnostics16183045 (PMC13605948; doi:10.3390/diagnostics16183045)
Supplement: Supplementary file 1 [file diagnostics-16-03045-s001.zip › Supplementary_Materials/Figures/figureS2_funnel.pdf]

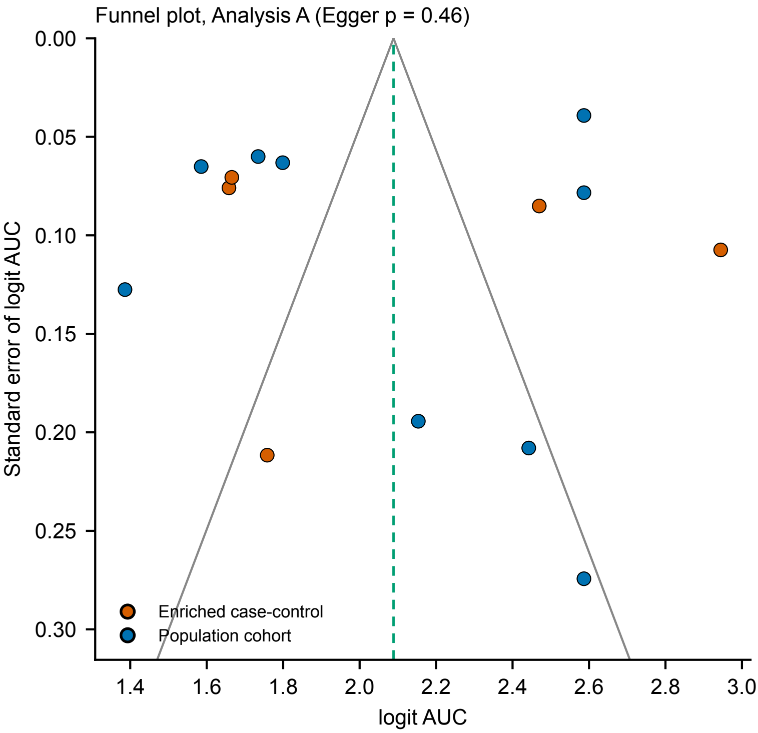
Funnel plot, Analysis A (Egger p = 0.46)
Standard error of logit AUC
0.00
0.05
0.10
0.15
0.20
0.25
0.30
logit AUC
1.4
1.6
1.8
2.0
2.2
2.4
2.6
2.8
3.0
Enriched case-control
Population cohort
