# Supplementary material for: Ten Years of Artificial Intelligence in Screening Mammography: A Systematic Review and Meta-Analysis of Diagnostic Accuracy and Clinical Implementation (Literature Published 2015–2025)
Source: Diagnostics (Basel). 2026 Sep 20;16(18):3045. doi: 10.3390/diagnostics16183045 (PMC13605948; doi:10.3390/diagnostics16183045)
Supplement: Supplementary file 1 [file diagnostics-16-03045-s001.zip › Supplementary_Materials/Protocol/Protocol_S1.pdf]

Protocol S1. Review protocol and analysis plan

Title. Ten Years of Artificial Intelligence in Screening Mammography: A Systematic Review and Meta-Analysis of Diagnostic Accuracy and Clinical Implementation

Registration. protocols.io, https://dx.doi.org/10.17504/protocols.io.q26g7q469lwz/v1

Reporting guidelines. PRISMA 2020, PRISMA-DTA, PRISMA-S

1. Objective

To determine, in women undergoing mammographic screening, how accurately deep-learning AI systems detect screen-relevant breast cancer when used standalone, and what happens to cancer detection, recall and reading workload when they are integrated into a screening programme; and to test whether reported accuracy has changed over the decade of literature searched.

2. Review question (PIRD)

Element	Specification
**P**opulation	Women undergoing screening mammography (digital mammography or digital breast tomosynthesis). Symptomatic and diagnostic populations excluded.
**I**ndex test	A deep-learning AI system producing an examination-level or lesion-level suspicion score, used standalone or integrated with radiologist reading.
**R**eference standard	Histopathology for screen-detected and clinically detected cancers, with a cancer-free follow-up interval of at least 12 months for negatives.
**D**iagnosis / target condition	Screen-relevant breast cancer, invasive or in situ, including interval cancers where reported.
Comparator	Radiologist reading as practised in the study's own setting.

Primary outcomes. Area under the ROC curve, and paired sensitivity and specificity, for standalone accuracy. Cancer detection rate ratio for AI-integrated versus standard reading.

Secondary outcomes. Recall rate ratio, positive predictive value of recall, interval cancer detection, screen-reading workload.

3. Eligibility criteria

Include: multi-reader multi-case studies; retrospective consecutive or case-control accuracy studies; external-validation studies; prospective paired-reader studies; before-and-after programme comparisons; randomised controlled trials. English language. Published 1 January 2015 to 31 December 2025.

Exclude: reviews, editorials and protocols without primary data; non-mammographic modalities; non-screening populations; pure risk prediction without a detection or triage endpoint; simulation or decision-

modelling studies without a real reading comparison; development studies evaluated only on public benchmark collections.

Grey literature: preprints indexed in Europe PMC and trial-registry records are screened under the same criteria. Conference abstracts are eligible only if they report extractable outcomes.

4. Information sources

PubMed; Europe PMC; ClinicalTrials.gov; forward and backward citation searching of all included studies and of previous systematic reviews. Full strategies with line-by-line yields are recorded in Table S1. Databases requiring a subscription we do not hold are not searched, and this is declared as a limitation.

5. Selection and extraction

Title-and-abstract screening is performed against the written rule set in Section S1, with every exclusion logged and the log published. All records surviving the first pass are retrieved in full and assessed independently in duplicate, with disagreements resolved by discussion and a third reviewer where discussion does not settle the question. Duplicate judgements are reconciled to consensus at the point of assessment; the protocol does not require the separate recording of disagreement counts, so no chance-corrected agreement statistic is produced.

Data are extracted in duplicate onto the form in Table S8. Discrepancies are reconciled against the source report.

5.1 Rules for selecting among multiple reported estimates

1. Where a study evaluates several AI systems without nominating one as primary, the **median-performing** system enters the main analysis; the best and worst are carried into a sensitivity analysis.
2. Where a study reports several independent cohorts, the **largest** enters the main analysis; the others are carried into a sensitivity analysis.
3. Where a study reports several thresholds, the one selected by a rule external to the evaluation data is preferred over one optimised on the evaluation data.

5.2 Cohort overlap

Multiple reports of the same trial are counted once, using the most complete report. Where two studies draw on the same underlying screening cohort, only one enters any single synthesis. Overlaps are declared in the manuscript and checked in the leave-one-out analysis.

6. Risk of bias and certainty

QUADAS-2 across all seven domains, with QUADAS-C where a comparative accuracy claim is made and PROBAST+AI informing the index-test domain where the study also developed the model. Two independent assessors, disagreements resolved by discussion, written rationale required for every judgement other than low risk. Certainty rated with GRADE for diagnostic test accuracy, one profile per synthesis outcome.

7. Analysis plan

Analysis A — standalone accuracy. AUCs transformed to the logit scale, standard errors transferred by the delta method, missing standard errors approximated from case and control counts by the Hanley–McNeil

method. Random effects with between-study variance by restricted maximum likelihood; confidence intervals with the Knapp–Hartung–Sidik–Jonkman correction. DerSimonian–Laird and Paule–Mandel reported as sensitivity analyses. Heterogeneity by Q, I^2 and τ^2. A 95% prediction interval is reported wherever $k \geq 5$. Leave-one-out for every study.

Heterogeneity. Subgroups pre-specified by design (enriched versus consecutive sampling) and by region, analysed only where $k \geq 3$. Random-effects meta-regression on publication year, design, and both, with R^2 reported.

Analysis A2 — bivariate model. Logit sensitivity and logit specificity modelled jointly (Reitsma type) by maximum likelihood, reporting the summary point, between-study standard deviations, their correlation and the summary diagnostic odds ratio. A hierarchical summary ROC curve is not fitted unless studies report multiple thresholds.

Analyses B and C. Rate ratios pooled on the log scale with the same estimator, stratified by evidence tier (prospective clinical evaluation; before-and-after programme cohort). The tiers are not combined. Within the prospective tier, individual trials are the primary presentation and the pooled estimate is secondary. Where a trial reports an interval accounting for a paired design, that interval is used; otherwise intervals are derived from independent Poisson variances and labelled as conservative.

Small-study effects. Egger's regression only where $k \geq 10$ in a stratum. No test is attempted below that.

Software. Python 3 (numpy, pandas, scipy). The analysis and figure scripts are published, and re-running them regenerates every reported estimate and figure from the extraction tables. No effect estimate is typed by hand into the manuscript or into the plotting code.

8. Amendments

The sections above describe the review as registered, except where marked. Everything listed here was decided after registration and should not be read as pre-specified; Section 2.9 of the manuscript carries the same list.

After the first round of peer review. Extension of the search beyond PubMed to Europe PMC, ClinicalTrials.gov and citation searching, and correction of the PubMed strategy. Change of the primary estimator from DerSimonian–Laird on the AUC scale to REML with Knapp–Hartung intervals on the logit-AUC scale. Addition of the selection rules in Section 5.1. Addition of Analyses A2 and C. Addition of subgroup and meta-regression analyses.

After the second round of peer review. Independent verification of the title-and-abstract screening stage against a second, separately specified procedure, which led to three further studies being included. Renaming of the evidence tiers in Analysis B to distinguish randomised and paired prospective designs from non-randomised implementation studies. Addition of sensitivity analyses for two newly identified cohort overlaps. Addition of an R reference implementation of the analyses.
