# Supplementary material for: Ten Years of Artificial Intelligence in Screening Mammography: A Systematic Review and Meta-Analysis of Diagnostic Accuracy and Clinical Implementation (Literature Published 2015–2025)
Source: Diagnostics (Basel). 2026 Sep 20;16(18):3045. doi: 10.3390/diagnostics16183045 (PMC13605948; doi:10.3390/diagnostics16183045)
Supplement: Supplementary file 1 [file diagnostics-16-03045-s001.zip › Supplementary_Materials/Tables/Section_S1_screening_rules.pdf]

Section S1. Screening rule set and screening log

S1.1 Why this section exists

Reviewers asked for inter-reviewer agreement statistics. At the first revision this review reported none. At the second revision the title-and-abstract stage was verified against an independently specified second screening procedure, and Section S1.6 reports that comparison, its agreement statistic and its consequences for the included set. The reason no statistic is reported for the full-text stage is given below.

Title-and-abstract screening of 1,743 records was carried out by an explicit, written rule set rather than by two independent human raters. A chance-corrected statistic is not defined for that stage, because there is one rater and it is deterministic. What we offer instead is complete auditability: the rule set is printed below, every exclusion is logged with the rule that produced it, and the log is published so that any reader can re-run the pass or disagree with it record by record.

Full-text assessment of all 157 reports that reached that stage — the 151 retrieved by the first pass and six reinstated afterwards — was performed independently in duplicate by two reviewers, with a third consulted where discussion did not resolve a disagreement. Those duplicate judgements were reconciled to consensus as the assessment proceeded rather than being recorded separately first, so the disagreement counts a kappa requires were not preserved and the statistic cannot be reconstructed. This is stated in Section 2.4 of the manuscript and again in its limitations.

S1.2 The rules, in the order they were applied

A record was excluded at the first rule it triggered, and the rule was recorded.

Rule	Excludes	Operationalisation
**E1**	Reviews and other non-primary reports	Publication type or title matching systematic review, meta-analysis, scoping review, narrative review, editorial, comment, letter, erratum, guideline, consensus statement, study protocol, umbrella review or bibliometric analysis
**E3**	Not mammography or tomosynthesis	Title and abstract containing no term matching `mammogra*`, `tomosynthes*`, `DBT` or `FFDM`
**E5**	No AI index test	No term matching deep learning, artificial intelligence, machine learning, neural network, convolutional, CNN, transformer, computer-aided detection or computer-aided diagnosis
**E2a**	Not a screening population	No term indicating a screening programme, screening cohort, population-based screening, organised screening or an asymptomatic population
**E4**	No detection or triage accuracy outcome	No term matching AUC, area under the curve, sensitivity, specificity, cancer detection rate, recall rate, detection rate, non-inferiority, reader study or false

		positive
**E2b**	Development study on public benchmark data	Reference to DDSM, CBIS, INbreast, MIAS, VinDr, an RSNA challenge dataset, a public or benchmark dataset, or framed purely as a data-augmentation, transfer-learning or segmentation method, *and* no reference to a screening programme, a population-based or consecutive cohort, or a prospective or randomised design

Records surviving all six rules were retrieved in full.

S1.3 What each rule removed

**Rule**	**Records excluded**
E2a, not a screening population	621
E3, not mammography or tomosynthesis	573
E4, no detection or triage accuracy outcome	144
E1, review or other non-primary report	111
E5, no AI index test	109
E2b, development study on public benchmark data	34
**Total excluded**	**1,592**
**Carried to full-text retrieval**	**151**

S1.4 Known behaviour of the rule set

Two properties are worth stating, because they bear on how much confidence the pass deserves.

The rules are deliberately permissive. Any record whose abstract mentions mammography, an AI method, a screening context and an accuracy outcome was retrieved in full, even where the abstract made clear it would probably be excluded later. That is why 133 of the 157 reports assessed at full text were excluded: the pass was tuned for sensitivity rather than precision, which is the correct trade-off when the cost of a missed study is much higher than the cost of reading one extra full text.

The rules operate on titles and abstracts only. A study reporting an eligible screening evaluation whose abstract omitted any mention of the screening setting would have been missed. Citation searching over 750 records (Section 2.3) was performed partly as a check on this failure mode and returned two additional eligible studies.

S1.5 Full-text exclusions

The 133 reports excluded after full-text assessment are listed individually, with their identifiers and the eligibility criterion each fails, in `fulltext_exclusions.csv` in Data S1. The tally printed in Figure 1 of the manuscript is generated from that file by `make_fulltext_exclusions.py`, so the reasons cannot disagree with the total.

Reason for exclusion at full text	Reports
Risk prediction or another non-detection target	29
Development or internal validation without an independent screening cohort	18
No extractable accuracy or programme outcome	18
Simulation or modelling of a reading strategy only	17
Review, editorial or protocol without primary data	14
Non-mammographic index modality	11
Cancer-only or interval-cancer case series without a screening denominator	9
Not a screening population	9
Superseded by, or not confirmably independent of, an included report	6
Index test not a deep-learning system	2
**Total**	**133**

Round-3 Reviewer 1 noted that the earlier version of this tally summed to 130 under a header of 127. Both numbers were wrong. The reasons above were re-derived at the third revision by re-assessing every report that reached full text against the criteria in Section 2.2 of the manuscript; the per-report reasons recorded during the original assessment had not been preserved, and this listing replaces them rather than reproducing them.

S1.6 Independent verification of the title-and-abstract screen

Why this was done

A single deterministic rule set is exactly reproducible but provides no independent check: there is one rater, so a chance-corrected agreement statistic is undefined and a systematic blind spot in the rules would go undetected. Reviewer 3 asked for independent verification of this stage. We built a second screening procedure, applied it to all 1,743 deduplicated records, compared the two, and adjudicated every disagreement.

How the second procedure differs

The two were written separately, and the second was not tuned to reproduce the first.

	Pass 1	Pass 2
Information source	Free text of title and abstract	NLM Medical Subject Headings and author keywords, with free text only where indexing is absent
Logic	Sequential exclusion cascade; the first rule to fire decides	Additive inclusion scoring; all four PIRD concepts must be present
Orientation	Exclusion lexicon	Inclusion lexicon, written independently

Non-primary designs	Publication-type field of the record	MeSH publication-type descriptors and title patterns

The full implementation is `screening_verification.py` in Data S1.

Agreement

	Pass 2 include	Pass 2 exclude	Total
**Pass 1 include**	143	8	151
**Pass 1 exclude**	242	1350	1592
**Total**	385	1358	1743

Raw agreement 85.7%. **Cohen's κ 0.47 (95% CI 0.41–0.53)**, which is moderate. The second procedure was substantially more inclusive, carrying 385 records to full text against 151.

Adjudication of the 250 discordant records

At the second revision this section stated that all 250 discordant records had been individually adjudicated by two reviewers. Round-3 Reviewer 1 observed that the adjudication file contained only 20 records. The observation is correct and the earlier wording overstated what had been done. What was done, and what the file now records in full, is a two-stage adjudication:

Stage 1 — record-level adjudication (230 records). Every discordant record was assessed against the eligibility criteria of Section 2.2 using its title, abstract and indexing terms. A record that fails a criterion unambiguously is recorded as ineligible together with the criterion it fails. This is one documented assessment per record. It is not duplicate independent human screening and is not offered as a substitute for it.

Stage 2 — full-text adjudication by two reviewers (20 records). Every record with any plausible route to inclusion — anything not excluded outright by a single criterion in stage 1 — was retrieved in full and adjudicated against the complete criteria by two reviewers, with disagreements resolved by discussion.

The complete listing is `screening_discordance.csv` and the decisions are `screening_adjudication.csv`, both in Data S1. Every one of the 250 rows carries a final decision, the criterion or reason behind it, and a `basis` column naming the stage that decided it, so the process can be checked rather than taken on trust. The outcome:

Adjudication	n
Eligible, newly included	3
Eligible but already in the review, having entered through citation searching	3
Ineligible	244

Of the 244 ineligible records, nine reported a cohort already represented in the review or one that could not be confirmed independent of it, including the two flagged under the cohort-overlap rule in Section 2.8. The

criteria behind the remainder are tabulated in the adjudication file; the largest groups are a non-mammographic index modality (51), a review, editorial or protocol without primary data (45), a development or internal-validation study (37), pure risk prediction (25) and a target condition other than screen-relevant breast cancer (20).

The three newly included studies are a nationwide German implementation study of 463,094 women, a multicentre multivendor validation of a triage algorithm, and a site-controlled evaluation of tomosynthesis screening with AI. Each is identified in the adjudication file with the reason it was missed.

What this says about pass 1

Round-3 Reviewer 1 asked us to define the denominator for this figure and to revise the calculation. The denominator is the number of eligible studies present in the 1,743 deduplicated database records. Twenty-four of the 26 included studies are in that record set; the other two, Kuhl 2024 and ScreenTrustCAD 2023, were retrieved outside the databases and cannot count for or against a database screening pass. Pass 1 identified 18 of those 24, **a sensitivity of 75%**, and missed six. The earlier figure of 17 of 20, or about 85%, was computed against the 22-study pool that preceded the three studies this verification added and the addition of McKinney 2020 to Table 1; it is withdrawn. The calculation is reproducible from `records_merged.json` and `screen_stage1.json` in Data S1.

Three of the six misses were recovered from reference lists at the first revision and three by this verification. All six were caused by the same failure mode: the record's abstract used a phrasing that the exclusion cascade did not recognise, and the cascade stops at the first rule that fires, so a single unmatched phrase removes a record permanently. An inclusion-scoring design does not have that property, which is why the second procedure recovered three of them.

We report this rather than presenting the screen as sound. It is the most important limitation of the review process, it is stated in Section 4.1 of the manuscript, and it is the reason we would not recommend a single-pass deterministic screen as a substitute for duplicate screening in future work.
