# Supplementary material for: Ten Years of Artificial Intelligence in Screening Mammography: A Systematic Review and Meta-Analysis of Diagnostic Accuracy and Clinical Implementation (Literature Published 2015–2025)
Source: Diagnostics (Basel). 2026 Sep 20;16(18):3045. doi: 10.3390/diagnostics16183045 (PMC13605948; doi:10.3390/diagnostics16183045)
Supplement: Supplementary file 1 [file diagnostics-16-03045-s001.zip › Supplementary_Materials/Tables/Table_S1_search_strategies.pdf]

Table S1. Full electronic search strategies (PRISMA-S)

All searches were executed on 11 August 2026 and restricted to records with a publication date between 1 January 2015 and 31 December 2025. Line-by-line yields are given so that the search can be re-executed and audited.

S1.1 PubMed (via NCBI Entrez, searched 11 August 2026)

Line	Query	Records
#1	`"Mammography"[MeSH] OR mammograph*[tiab] OR "digital breast tomosynthesis"[tiab] OR tomosynthes*[tiab] OR "Breast Neoplasms/diagnostic imaging"[MeSH]`	66,575
#2	`"Artificial Intelligence"[MeSH] OR "Deep Learning"[MeSH] OR "Machine Learning"[MeSH] OR "Neural Networks, Computer"[MeSH] OR "deep learning"[tiab] OR "artificial intelligence"[tiab] OR "machine learning"[tiab] OR "neural network*"[tiab] OR convolutional[tiab] OR "computer-aided detection"[tiab] OR "computer-aided diagnosis"[tiab] OR "computer aided"[tiab]`	589,522
#3	`"Mass Screening"[MeSH] OR "Early Detection of Cancer"[MeSH] OR screening[tiab] OR "diagnostic accuracy"[tiab] OR "Sensitivity and Specificity"[MeSH] OR "cancer detection rate"[tiab] OR triage[tiab] OR "reader study"[tiab] OR "second reader"[tiab]`	1,661,869
#4	`#1 AND #2 AND #3`	2,921
#5	`#4 AND ("2015/01/01"[PDAT] : "2025/12/31"[PDAT])`	1,822
#6	`#5 AND Humans[MeSH]`	1,527
#7	`#6 AND English[LA]`	**1,503**

Twenty-four non-English records were removed at line #7. Their titles were inspected; none described a screening-population evaluation of AI that was not already represented by an English-language report of the same programme.

Change from the strategy reported in the original submission

The submitted strategy contained `"Tomography, X-Ray Computed"[MeSH]`, which indexes computed tomography and not breast tomosynthesis, and contained no free-text term for tomosynthesis or DBT. That strategy returned 4,043 records, inflated by computed-tomography literature, while missing tomosynthesis-

specific reports. The corrected strategy above adds `tomosynthes*[tiab]`, `"digital breast tomosynthesis"[tiab]`, `mammograph*[tiab]` and `"Breast Neoplasms/diagnostic imaging"[MeSH]`, and removes the computed-tomography term. We thank the reviewer who identified this error.

S1.2 Europe PMC (REST API, searched 11 August 2026)

Europe PMC indexes MEDLINE together with content that PubMed does not cover, including preprint servers, Agricola, and European doctoral theses. It was searched to extend coverage beyond PubMed where subscription databases were not available to us.

Line	Query	Records
#1	`TITLE_ABS:mammograph* OR TITLE_ABS:tomosynthes* OR TITLE_ABS:"digital breast tomosynthesis"`	40,582
#2	`TITLE_ABS:"artificial intelligence" OR TITLE_ABS:"deep learning" OR TITLE_ABS:"machine learning" OR TITLE_ABS:"neural network" OR TITLE_ABS:convolutional OR TITLE_ABS:"computer-aided detection" OR TITLE_ABS:"computer-aided diagnosis"`	499,880
#3	`TITLE_ABS:screening OR TITLE_ABS:"diagnostic accuracy" OR TITLE_ABS:"cancer detection rate" OR TITLE_ABS:triage OR TITLE_ABS:"reader study" OR TITLE_ABS:"second reader"`	1,065,469
#4	`#1 AND #2 AND #3`	1,338
#5	`#4 AND FIRST_PDATE:[2015-01-01 TO 2025-12-31]`	**914**

Breakdown of line #5 by source: MEDLINE 837, preprint servers 70, European theses 3, Agricola 3, other 1. The preprint and thesis records are not indexed in PubMed and represent the grey-literature component of the search. Two preprints were carried through to full-text assessment; one, the interim analysis of AI-STREAM, was superseded by the peer-reviewed report and is cited in its published form.

S1.3 ClinicalTrials.gov (API v2, searched 11 August 2026)

Query	Records
`(mammography OR mammographic OR tomosynthesis) AND ("artificial intelligence" OR "deep learning" OR "machine learning" OR "computer-aided detection") AND screening`	**51**

Registry records were screened to identify completed but unpublished evaluations and ongoing trials. None contributed results to the quantitative synthesis. Ongoing trials identified in this way are listed in Table S5 and are discussed in the section on future research.

S1.4 Citation searching

The studies included from the database searches and the five previous systematic reviews were used as seeds. Both the works citing each seed and the works cited by each seed were retrieved through the Europe PMC citation network on 11 August 2026 and restricted to MEDLINE records published between 2015 and 2025.

Step	Records
Unique records in the citation network, not already retrieved	750
Passing the title-level concept filter (mammography AND artificial intelligence AND screening)	7
Retrieved and assessed in full	7
Excluded: simulation only	2
Excluded: development study on public datasets	1
Excluded: survey of preferences	1
Excluded: further report of a trial already included	2
**Included**	**1**

The one study added by this route (Kühl 2024) is a population-wide Danish evaluation covering 249,402 screening examinations and 2,033 cancers, which contributed to Analysis A2. That a search of 750 citation-network records yielded exactly one additional eligible study is the strongest evidence we can offer that the database searches were close to saturated for this question.

S1.5 Databases that were not searched

Embase, Scopus, Web of Science, IEEE Xplore and the Cochrane Library were not searched, because none is available under our institutional subscriptions and none offers a programmatic interface that would allow the search to be documented reproducibly. This is stated as a limitation in the manuscript. Three considerations bound the likely consequence:

1. Cochrane CENTRAL is populated chiefly from MEDLINE, Embase and trial registries. Two of these three streams were searched directly.
2. IEEE Xplore and Scopus add engineering and computer-science literature. Studies indexed only there almost never report a consecutive screening population with a histopathological reference standard and cancer-free follow-up, which is the eligibility criterion that removed the large majority of records at the title-and-abstract stage.

3. Citation searching over 750 records seeded from the included studies and the five previous syntheses (Section S1.4) returned a single additional eligible study. If a substantial body of eligible work were sitting outside PubMed and Europe PMC, the citation network of this literature would be expected to surface more of it than that.

S1.6 A note on completeness, added at the second revision

The searches described above were not changed at the second revision, but the *screening* of their output was independently verified (Section S1.6). That verification recovered three eligible studies the first screening pass had missed, all of them present in the retrieved records rather than absent from the search. The gap was therefore in screening, not in retrieval, and the corrected total is 26 included studies.

S1.7 Reproducing the search

The record identifiers, the deduplication log and the screening decisions for all 1,743 unique records are provided as machine-readable files in the data package accompanying this article (`records_merged.json`, `screen_stage1.json`, `citation_chase.json`). Running `analysis/meta_analysis.py` and `analysis/make_figures.py` regenerates every number and every figure in the manuscript from the extraction tables.
