# Supplementary material for: Ten Years of Artificial Intelligence in Screening Mammography: A Systematic Review and Meta-Analysis of Diagnostic Accuracy and Clinical Implementation (Literature Published 2015–2025)
Source: Diagnostics (Basel). 2026 Sep 20;16(18):3045. doi: 10.3390/diagnostics16183045 (PMC13605948; doi:10.3390/diagnostics16183045)
Supplement: Supplementary file 1 [file diagnostics-16-03045-s001.zip › Supplementary_Materials/Tables/Table_S2_QUADAS2.pdf]

Table S2. QUADAS-2 risk-of-bias and applicability judgements

Assessed independently by two reviewers with disagreements resolved by discussion, for all 26 included studies. QUADAS-C was applied where a study made a comparative accuracy claim. Ratings: Low, Unclear, High.

RoB, risk of bias; App., applicability concerns.

**Study**	**Analysis**	**RoB: patient selection**	**RoB: index test**	**RoB: reference standard**	**RoB: flow and timing**	**App.: patient selection**	**App.: index test**	**App.: reference standard**
Rodriguez-Ruiz (2019)	A	High	Low	Low	Unclear	High	Low	Low
Salim (2020)	A	High	Low	Low	Low	High	Low	Low
Schaffter (2020)	A	Low	Unclear	Low	Low	Low	Unclear	Low
Kizildag Yirgin (2022)	A	High	High	Low	Low	High	Low	Low
Romero-Martin (2022)	A	Low	Low	Low	Low	Low	Low	Low
Hsu (2022)	A	Low	Low	Low	Low	Low	Low	Low
Marinovich (2023)	A	Low	Low	Low	Low	Low	Low	Low
Riveira-Martin (2023)	A	Low	Low	Unclear	Unclear	Low	Low	Unclear
Kwon (2024)	A	Low	Low	Low	Low	Low	Low	Unclear
Seker (2024)	A	Unclear	High	Low	High	Low	Low	Low
Larsen (2024)	A	Low	Low	Low	Low	Low	Low	Low
Graham-Knight (2025)	A	Low	Low	Low	Low	Low	Low	Low
Yamaguchi (2025)	A	High	High	Low	Low	High	Low	Low
MASAI (2025)	B	Low	Low	Low	Low	Low	Low	Low
ScreenTrustCAD (2023)	B	Low	Low	Low	Low	Low	Low	Low
AI-STREAM (2025)	B	Low	Low	Low	Unclear	Low	Low	Low
Lauritzen (2024)	B	Unclear	Low	Low	Low	Low	Low	Low
Elias-Cabot (2024)	B	Unclear	Low	Unclear	Unclear	Low	Low	Unclear
Nepute (2025)	B	High	Low	Unclear	High	Unclear	Low	Unclear

Hickman (2024)	A2	Low	Low	Low	Low	Low	Low	Low
Kuhl (2024)	A2	Low	Low	Low	Low	Low	Low	Low
Sandler Rahat (2025)	B	High	Low	Unclear	High	Unclear	Low	Unclear
Retson (2022)	A	High	Low	Low	Unclear	High	Low	Low
PRAIM (2025)	B	High	Low	Low	Low	Low	Low	Low
Letter (2023)	B	High	Low	Unclear	Unclear	Unclear	Low	Unclear
McKinney (2020)	Narrative	High	Unclear	Low	Unclear	High	Low	Low

Rationale for each judgement

Rodriguez-Ruiz (2019). Cancer-enriched multi-reader datasets assembled for a reader study; case mix does not match a consecutive screening population. Index test read blinded to outcome. Reference standard histopathology. Interval between index test and reference standard not fully reported.

Salim (2020). Case-control sampling with all cancers and a random control sample; spectrum is enriched. Threshold pre-set at the mean first-reader specificity and applied blind. Histopathology plus two-year cancer-free follow-up.

Schaffter (2020). Consecutive population screening cohort. Algorithms were selected as the top performers of a challenge leaderboard on partly overlapping data, so the index test may be optimistically selected. Registry-linked outcomes.

Kizildag Yirgin (2022). Small case-control sample (110 cancers vs 101 controls) drawn from a screening programme archive. The decision threshold was derived from the same data by Youden optimisation, so accuracy is likely optimistic.

Romero-Martin (2022). Consecutive screening examinations from a screening trial; paired DM and DBT read independently; histopathology with follow-up for interval cancers.

Hsu (2022). Consecutive screening cohort from a distributed academic programme with registry-confirmed one-year outcomes; pre-specified ensemble applied without retraining.

Marinovich (2023). Consecutive population screening cohort with registry linkage for interval cancers; vendor-recommended threshold applied prospectively.

Riveira-Martin (2023). Population screening cohort scored by the AI system. Reference-standard ascertainment and follow-up interval are only briefly described.

Kwon (2024). Consecutive initial screening examinations with national registry linkage. Cancers within twelve months of screening were used as the reference standard; a twelve-month window may misclassify some later interval cancers.

Seker (2024). Programme cohort with substantial data loss (5,136 of 22,621 mammograms analysed). The threshold was optimised on the same data by Youden index.

Larsen (2024). Very large consecutive national screening cohort with complete registry follow-up; continuous AI score analysed without threshold selection on the same data.

Graham-Knight (2025). Consecutive provincial screening cohort with one- and two-year registry follow-up; commercial algorithm applied without local retraining.

Yamaguchi (2025). Mixed screening and diagnostic mammograms with a deliberately balanced cancer proportion; the test set came from the same multi-institutional collection used for development, so the evaluation is internal rather than external.

MASAI (2025). Randomised parallel-group allocation within a national screening programme; outcome assessors blinded; complete follow-up for the reported secondary endpoints.

ScreenTrustCAD (2023). Prospective consecutive enrolment with a paired design; all reading arms applied to the same women; histopathology reference standard.

AI-STREAM (2025). Prospective multicentre enrolment in a national screening programme; this is a pre-planned preliminary analysis and the full follow-up window is not yet complete.

Lauritzen (2024). Before-after comparison of two consecutive cohorts; screening interval differed between periods, so temporal confounding cannot be excluded.

Elias-Cabot (2024). Before-after comparison in a single programme; follow-up for interval cancers in the post-implementation period was short.

Nepute (2025). Before-after comparison across non-contiguous periods with only four readers and an interrupted post-implementation window; the comparator was conventional CAD rather than unaided reading.

Hickman (2024). Consecutive full-field digital mammograms from two UK screening centres with three-year follow-up; the operating threshold was fixed in advance to match a single human reader.

Kuhl (2024). Entire regional screening population with 24-month follow-up and registry-confirmed interval cancers; thresholds matched to first-reader sensitivity and specificity rather than optimised on the evaluation data. Draws on the same Region of Southern Denmark cohort as an earlier report, so the two are not both used in any single synthesis.

Sandler Rahat (2025). Retrospective audit comparing single calendar years before and after AI integration, with unequal and partly unreported denominators; contributes to the narrative synthesis only.

Retson (2022). Enriched case-control sample of 400 cancers and 855 controls assembled across institutions and vendors; the operating point was the vendor default rather than one fitted to these data. Interval between index test and reference standard not reported.

PRAIM (2025). Very large prospective multicentre implementation in an organised programme with registry-confirmed outcomes, but radiologists chose voluntarily whether to use AI, so allocation to the AI arm was self-selected and reader-level confounding cannot be excluded.

Letter (2023). Concurrent comparison of one AI-equipped site against two sites without AI, with five readers and no adjustment for site or reader case mix; follow-up for interval cancers not reported.

McKinney (2020). A representative United Kingdom screening dataset paired with an enriched United States dataset; the evaluating team also developed the model, and the reported accuracy is expressed as a difference from readers rather than as an estimate with an interval, so the study contributes to the narrative synthesis only.
