# Supplementary material for: Ten Years of Artificial Intelligence in Screening Mammography: A Systematic Review and Meta-Analysis of Diagnostic Accuracy and Clinical Implementation (Literature Published 2015–2025)
Source: Diagnostics (Basel). 2026 Sep 20;16(18):3045. doi: 10.3390/diagnostics16183045 (PMC13605948; doi:10.3390/diagnostics16183045)
Supplement: Supplementary file 1 [file diagnostics-16-03045-s001.zip › Supplementary_Materials/Tables/Table_S3_GRADE_DTA.pdf]

Table S3. GRADE certainty of evidence for each synthesis outcome

Certainty was rated for each outcome by two reviewers working independently, with disagreements settled by discussion. Diagnostic-accuracy outcomes start at high certainty when they derive from cross-sectional or cohort studies that enrol a consecutive or random sample of a relevant population, and at low certainty when they derive from case-control sampling. Programme-level outcomes were rated using the standard GRADE domains for comparative effectiveness.

Outcome 1 — Standalone AI detection accuracy (pooled AUC, k = 14)

Domain	Rating	Reason
Study design	Cross-sectional accuracy studies, 9 consecutive cohorts and 5 case-control	Starting point: moderate, because more than a third of the pool used enriched sampling
Risk of bias	Serious limitation (−1)	5 of the 14 studies in this pool were judged at high risk in patient selection and 3 at high risk in the index-test domain, mainly because the decision threshold was optimised on the same data (Table S2)
Indirectness	Serious limitation (−1)	The index test differs across studies in vendor, version and operating point; two studies mixed screening with diagnostic examinations; the reference standard follow-up window ranged from 12 to 36 months
Inconsistency	Very serious limitation (−2)	I^2 = 97.4%; the 95% prediction interval, 0.731 to 0.960, spans the range from clearly inferior to clearly superior to a radiologist. Neither publication year nor study design explained the dispersion in meta-regression
Imprecision	No limitation	The pooled interval is narrow (0.858 to 0.915) and more than 1.2 million examinations contribute
Publication bias	No limitation	Egger test p = 0.66 with k = 14; the funnel plot (Figure S2) is symmetrical
**Certainty**	**Very low**	

What this means. We are very uncertain about the pooled standalone accuracy of AI in screening mammography. The estimate should be read as a description of what has been reported, not as a performance figure that a given system would achieve in a given programme.

Outcome 2 — Summary sensitivity and specificity (bivariate model, k = 9)

Domain	Rating	Reason
Study design	Six cohorts, three case-control	Starting point: moderate
Risk of bias	Serious limitation (−1)	Three of the nine studies selected the operating point on the same data used for evaluation
Indirectness	Very serious limitation (−2)	The threshold rule differs fundamentally between studies: some matched radiologist specificity, others optimised the Youden index, others used the vendor default. Sensitivity and specificity are therefore not estimates of the same quantity
Inconsistency	Serious limitation (−1)	Between-study SD was 0.62 on the logit sensitivity scale and 0.93 on the logit specificity scale, corresponding to specificities from roughly 76% to 98%
Imprecision	No limitation	5,550 cancers contribute
Publication bias	Undetected	Too few studies to assess
**Certainty**	**Very low**	

Outcome 3 — Cancer detection rate, randomised and paired prospective evidence (k = 3)

Domain	Rating	Reason
Study design	One randomised trial, one prospective paired-reader trial, one prospective cohort	Starting point: high
Risk of bias	No limitation	All three enrolled consecutively within organised programmes with registry-confirmed outcomes
Indirectness	Serious limitation (−1)	The comparator differs across the three trials: standard double reading, two-radiologist double reading, and unaided single reading. Only two European programmes and one Asian programme are represented
Inconsistency	Serious limitation (−1)	$I^2 = 69.9\%$; the point estimates range from 1.04 to 1.29, which is the difference between non-inferiority and a clinically

		meaningful gain
Imprecision	Serious limitation (−1)	The pooled interval, 0.83 to 1.55, includes both a reduction and a large increase in detection
Publication bias	Undetected	Too few studies to assess
**Certainty**	**Low** for the pooled estimate; **moderate** for the MASAI trial alone	

What this means. Considered on its own, the MASAI randomised trial provides moderate-certainty evidence that AI-supported reading increases cancer detection in a Swedish programme (rate ratio 1.29, 1.09 to 1.51), downgraded once for indirectness because a single programme, a single AI system and a single reading protocol were studied. The pooled estimate across the three prospective studies carries low certainty and should not be quoted as the expected effect of "adding AI".

Outcome 4 — Cancer detection rate, non-randomised implementation studies (k = 5)

**Domain**	**Rating**	**Reason**
Study design	One prospective opt-in implementation study, three before-and-after comparisons and one concurrent site-controlled comparison	Starting point: low
Risk of bias	Very serious limitation (−2)	Secular trends, changes in screening interval and reader composition are uncontrolled; one study used conventional CAD rather than unaided reading as the comparator; and in the largest study, which contributes most of the weight, radiologists chose voluntarily whether to use AI, so the comparison is subject to reader self-selection
Indirectness	No further limitation	The populations and outcomes are directly relevant
Inconsistency	No limitation	$I^2 = 19.0\%$; point estimates ranged from 1.18 to 1.65 but the intervals overlap substantially
Imprecision	No limitation	Pooled interval 1.08 to 1.37, based on nearly 700,000 women
Publication bias	Suspected	Programmes that observed no change may be less likely to publish an audit
**Certainty**	**Very low**	

Outcome 5 — Recall rate (k = 6)

Domain	Rating	Reason
Study design	One randomised trial and five non-randomised implementation studies	Starting point: low
Risk of bias	Serious limitation (−1)	Dominated by non-randomised comparisons
Indirectness	No limitation	Recall is measured consistently across programmes
Inconsistency	Very serious limitation (−2)	$I^2 = 90.9\%$; the direction of effect is not consistent, with ratios from 0.79 to 1.13
Imprecision	Serious limitation (−1)	Pooled interval 0.81 to 1.12
Publication bias	Undetected	Too few studies to assess
**Certainty**	**Very low**	

Outcome 6 — Screen-reading workload

Not meta-analysed. Reductions were reported as 44.2% (MASAI), 62.6% (Danish simulation), 30.0% to 44.8% (multi-vendor simulation), 41.4% (BreastScreen Western Australia) and 38.3% to 43.7% (ARIES). These figures depend entirely on the triage threshold chosen by each programme and are not a property of the AI systems, so pooling them would be misleading. Certainty was not rated for a quantity that is set by the user rather than measured.

Summary

Outcome	k	Estimate (95% CI)	Certainty
Standalone AI AUC	14	0.890 (0.858–0.915)	Very low
Summary sensitivity	9	73.3% (64.5–80.6)	Very low
Summary specificity	9	92.4% (86.8–95.8)	Very low
CDR ratio, randomised and paired prospective	3	1.13 (0.83–1.55)	Low
CDR ratio, MASAI trial alone	1	1.29 (1.09–1.51)	Moderate
CDR ratio, non-randomised implementation	5	1.22 (1.08–1.37)	Very low
Recall rate ratio	6	0.95 (0.81–1.12)	Very low
