# Supplementary material for: Ten Years of Artificial Intelligence in Screening Mammography: A Systematic Review and Meta-Analysis of Diagnostic Accuracy and Clinical Implementation (Literature Published 2015–2025)
Source: Diagnostics (Basel). 2026 Sep 20;16(18):3045. doi: 10.3390/diagnostics16183045 (PMC13605948; doi:10.3390/diagnostics16183045)
Supplement: Supplementary file 1 [file diagnostics-16-03045-s001.zip › Supplementary_Materials/Tables/Table_S4_extraction_tables.pdf]

Table S4a–d. Complete extraction tables

These are the files read by `meta_analysis.py` and `meta_analysis.R`. Every estimate in the manuscript is computed from them; nothing is typed by hand into the prose or the plotting code. Each row carries the PubMed identifier and DOI of its source report.

Table S4a. Analysis A — standalone accuracy

study_id	short_label	year	country	region	design_class	design_detail	modality	setting	n_exams	n_cancers	n_controls	ai_system	auc	auc_lci	auc_uci	ci_source	sens	spec	comparator_note	multi_system_rule	pmid	doi
Rodriguez-Ruiz 2019	Rodriguez-Ruiz (2019)	2019	Seven countries	Multinational	Enriched	Multi-reader multi-case reader study	DM	Enriched reader-study datasets	2652.0	653.0	1999.0	Transpara	0.84	0.82	0.86	reported			Mean of 101 radiologists AUC 0.814	Single system	30834436	10.1093/jnci/djy222
Salim 2020	Salim (2020)	2020	Sweden	Europe	Enriched	Retrospective case-control nested in a screening cohort	DM	Karolinska screening cohort 2008-2015	8805.0	739.0	8066.0	AI-2 (median of three vendors)	0.922	0.91	0.934	reported	67.0	96.6	First-reader radiologist sensitivity 77.4% at specificity 96.6%	Median of three systems (AI-1 0.956; AI-2 0.922; AI-3 0.920)	32852536	10.1001/jamaoncol.2020.3321
Schaffer 20	Schaffer (20	2020	USA	North Americ	Cohort	Retrospective	DM	Kaiser Permanen	144231.0	952.0	143279.0	Top-ranked	0.858			Hanley-M			No single algorith	Largest cohort (US)	32119094	10.1001/jamanetworkopen.2020.0265

20	20)			a		cohort (DREAM challenge)		te Washington screening				DREAM algorithm				cNeil			m exceeded radiologists; AI-plus-radiologist ensemble 0.942	primary; Swedish cohort AUC 0.903 in sensitivity analysis		
Kizildag Yirgin 2022	Kizildag Yirgin (2022)	2022	Turkiye	Other	Enriched	Retrospective case-control within an organised screening programme	DM	Bahcesehir 10-year screening programme	211.0	110.0	101.0	Commercial AI system	0.853	0.801	0.905	reported	72.8	88.3	Radiologist cancer detection 67.3% vs AI 72.7%	Single system	35060413	10.1177/15330338221075172
Romero-Martin 2022	Romero-Martin (2022)	2022	Spain	Europe	Cohort	Retrospective evaluation of a con	DM	Tomosynthesis Cordoba Screening Tri	15999.0	113.0	15886.0	Transpara	0.93	0.89	0.96	reported			Paired DBT arm AUC 0.94 (0.91-0.97	DM arm primary; DBT arm in modality subgroup	34904872	10.1148/radiol.211590

						secutive screening cohort		al 2015-2016											)			
Hsu 2022	Hsu (2022)	2022	USA	North America	Cohort	Retrospective external validation of a DREAM ensemble	DM	UCLA Athena Breast Health Network 2010-2020	37317.0			DREAM challenge ensemble model	0.85	0.84	0.87	reported	81.3	92.5	Radiologist sensitivity 82.6% specificity 93.0%	Ensemble designated primary by the authors	36409497	10.1001/jamanetworkopen.2022.42343
Marinovich 2023	Marinovich (2023)	2023	Australia	Oceania	Cohort	Retrospective external validation in a population screening cohort	DM	BreastScreen Western Australia	108970.0	760.0	108210.0	Commercial AI algorithm	0.83			Hanley-McNeil	67.0	81.0	Radiologist AUC 0.93; radiologist sensitivity 68% specificity 97%	Single system	36863255	10.1016/j.ebiom.2023.104498

Riveira-Martin 2023	Riveira-Martin (2023)	2023	Spain	Europe	Cohort	Retrospective population-based screening cohort	DM	Regional population screening programme				Commercial AI system	0.92	0.89	0.95	reported			Performance invariant to vendor and acquisition parameters	Single system	37082509	10.1117/1.JMI.10.5.051807
Kwon 2024	Kwon (2024)	2024	South Korea	Asia	Cohort	Retrospective population screening cohort	DM	Korean screening mammography 2009-2020	89855.0	143.0	89712.0	Lunit INSIGHT MMG	0.8	0.76	0.84	reported	67.1	93.0	Radiologist AUC 0.74 (0.70-0.78)	Single system	38649889	10.1186/s13058-024-01821-w
Seker 2024	Seker (2024)	2024	Turkiye	Other	Cohort	Retrospective biennial two-reader screening programme	DM	10-year biennial screening programme	5136.0	105.0	5031.0	Commercial AI system	0.896	0.861	0.932	reported	72.4	92.9	Two-reader programme comparator	Single system	38388718	10.1007/s00330-024-10661-3
Larse	Larse	20	Nor	Europ	Coho	Retros	DM	BreastS	6616	4917	6567	Tran	0.	0.9	0.9	rep			Screen-	Screen-	3859	10.1148/ryai.23037

n 2024	n (2024)	24	way	e	rt	pective population screening cohort		creen Norway 2004-2018	95.0	.0	78.0	spara	93	2	3	orted			detected cancers alone AUC 0.97	detected plus interval cancers as primary target condition	7784	5
Graham-Knight 2025	Graham-Knight (2025)	2025	Canada	North America	Cohort	Retrospective population screening cohort	DM	BC Cancer Breast Screening Programme 2019-2020	136700.0			Commercial AI algorithm	0.93	0.92	0.94	reported	89.4		Radiologist sensitivity 92.6% at 1-year follow-up	Single system	40072215	10.1148/ryai.240287
Yamaguchi 2025	Yamaguchi (2025)	2025	Japan	Asia	Enriched	Retrospective multi-institutional development and testing (63	DM	Mixed screening and diagnostic mammograms	2059.0	500.0	1559.0	DLADS (SE-ResNet)	0.841	0.822	0.859	reported	83.5	84.7	Pre-specified 80% sensitivity and specificity target	Single system	40608200	10.1007/s12282-025-01741-3

						sites)																
Retson 2022	Retson (2022)	2022	USA	North America	Enriched	Retrospective multicentre multivendor case-control validation	DM	Screening mammograms from multiple US institutions	1255.0	400.0	855.0	Commercial triage algorithm (cmTriage)	0.95	0.94	0.96	reported	93.0	76.3	Benchmarked against Breast Cancer Surveillance Consortium reader performance	Single system	38416951	10.1093/jbi/wbac046

Table S4b. Analysis A2 — reconstructed 2 × 2 tables

TP, FN, FP and TN were reconstructed from the reported sensitivity, specificity and denominators where a 2 × 2 table was not printed in the source report.

study_id	short_label	year	design_class	ai_system	threshold_rule	n_cancers	n_controls	sens	spec	tp	fn	fp	tn	pmid
Salim 2020	Salim (2020)	2020	Enriched	AI-2 (median of three)	Cut point set at mean first-reader specificity (96.6%)	739	8066	67.0	96.6	495	244	274	7792	32852536
Kizildag Yirgin 2022	Kizildag Yirgin (2022)	2022	Enriched	Commercial AI system	Youden-optimised risk-score cut-off (34.5%)	110	101	72.8	88.3	80	30	12	89	35060413
Marinovich 2023	Marinovich (2023)	2023	Cohort	Commercial AI algorithm	Prospective vendor-recommended threshold	760	108210	67.0	81.0	509	251	20560	87650	36863255

Kwon 2024	Kwon (2024)	2024	Cohort	Lunit INSIGHT MMG	Probability-of-malignancy cut-off 10%	143	89712	67.1	93.0	96	47	6280	83432	38649889
Seker 2024	Seker (2024)	2024	Cohort	Commercial AI system	Youden-optimised cut-off (30.44)	105	5031	72.4	92.9	76	29	357	4674	38388718
Hickman 2024	Hickman (2024)	2024	Cohort	DL-3 (median of three algorithms by sensitivity)	Preset specificity matched to a single human reader	760	25962	58.9	97.9	448	312	545	25417	39560480
Yamaguchi 2025	Yamaguchi (2025)	2025	Enriched	DLADS (SE-ResNet)	Heatmap concentration-gradient cut-off 15%	500	1559	83.5	84.7	418	82	239	1320	40608200
Kuhl 2024	Kuhl (2024)	2024	Cohort	Commercial AI system	Cut point matched to mean first-reader specificity	2033	247369	62.6	97.7	1273	760	5690	241679	37938386
Retson 2022	Retson (2022)	2022	Enriched	Commercial triage algorithm (cmTriage)	Vendor default operating point	400	855	93.0	76.3	372	28	203	652	38416951

Table S4c. Analyses B and C — programme-level outcomes

study_id	short_label	year	country	evidence_tier	design	ai_role	comparator	n_intervention	n_control	cancers_intervention	cancers_con	cdr_intervention	cdr_contro	rr_reporte	rr_lci_reported	rr_uci_reporte	recall_intervention_pct	recall_control_pct	recall_rr_reported	recall_lci_reported	recall_uci_reported	workload_reduction_pct	include_cd	exclusion_reason	pmid	doi

								n			trol		l	d		d							r			
MASAI2025	MASAI(2025)	2025	Sweden	Randomised or paired prospective	Randomised controlled parallel-group non-inferiority trial	AI triage plus detection support	Standard double reading	53043.0	52872.0	338.0	262.0	6.4	5.0	1.29	1.09	1.51	2.09	1.94				44.2	yes		39904652	10.1016/S2589-7500(24)00267-X
ScreenTrustCAD	ScreenTrustCAD	2023	Sweden	Randomised or	Prospectivepo	AI replacing	Double reading b	55581.0	55581.0	261.0	250.0	4.7	4.5	1.04	1.0	1.09							yes		37690911	10.1016/S2589-7500(23)0015

2023	(2023)			paired prospective	population-based paired-reader non-inferiority study	one of two readers	y two radiologists																			3-X
AI-STREAM2025	AI-STREAM(2025)	2025	South Korea	Randomised or paired prospec	Prospective multicentre paired co	AI-CAD concurrent supp	Single reading without AI	24543.0	24543.0	140.0	123.0	5.7	5.01	1.138									yes		40050619	10.1038/s41467-025-57469-3

				tive	hort study	ort in single reading																				
Lauritzen 2024	Lauritzen (2024)	2024	Denmark	Non-randomised implementation	Retrospective before-after comparison of consecutiv	AI triage plus decision support	Double reading before AI implementation	58246.0	60751.0	480.0	423.0	8.24	6.96				2.46	3.09					yes		38832880	10.1148/radiol.232479

					e screening cohorts																					
Elias-Cabot 2024	Elias-Cabot (2024)	2024	Spain	Non-randomised implementation	Retrospective before-after comparison of consecutive screeni	AI concurrent support for double reading	Double reading before AI implementation	11998.0	11998.0	108.0	70.0	9.0	5.8				6.1	5.4					yes		37975920	10.1007/s00330-023-10426-4

					ng cohorts																					
Nepute 2025	Nepute (2025)	2025	USA	Non-randomised implementation	Retrospective before-after comparison of DBT screening interpretation	AI concurrent support for DBT reading	Conventional CAD before AI implementation	64007.0	103222.0	39.0	38.0	6.1	3.7				6.5	8.2					yes		40473527	10.1016/j.clbc.2025.05.007

					ns																					
SandlerRahat2025	SandlerRahat(2025)	2025	Israel	Non-randomised implementation	Retrospective audit of screening before and after AI integration	AI-CAD concurrent support	Screening before AI integration	68003.0	24373.0			9.3	6.2										no	Denominators for the reported year-specific detection rates are not separately reported; d	41227281	10.3390/jcm14217886

																								escribed narratively only		
PRAIM2025	PRAIM (2025)	2025	Germany	Non-randomised implementation	Prospective multicentre real-world implementation study	AI-supported double reading, radiologist	Standard double reading without AI	260739.0	202355.0	1747.0	1153.0	6.7	5.7	1.176	1.057	1.308	3.74	3.83	0.975	0.935	1.017		yes		39775040	10.1038/s41591-024-03408-6

						opts in																				
Letter 2023	Letter (2023)	2023	USA	Non-randomised implementation	Concurrent site-controlled comparison of DBT screening interpretations	AI concurrent support for DBT reading	Sites reading DBT without AI over the same period					7.3	5.9	1.3	0.9	1.7	11.7	11.8	1.0	0.8	1.3		yes		38416890	10.1093/jbi/wbad015

Table S4d. Reporting completeness behind Figure 2, panel B

One row per included study, so each judgement in the figure can be checked.

study_id	short_label	system_version	architecture	density_subgroup	external_to_development	interval_cancers	sens_and_spec	reader_interaction	prospective_or_randomised
Rodriguez-Ruiz 2019	Rodriguez-Ruiz (2019)	yes	no	no	yes	no	no	yes	no
Salim 2020	Salim (2020)	no	no	no	yes	yes	yes	yes	no
Schaffter 2020	Schaffter (2020)	no	yes	no	no	no	yes	yes	no
Kizildag Yirgin 2022	Kizildag Yirgin (2022)	no	no	no	yes	yes	yes	no	no
Romero-Martin 2022	Romero-Martin (2022)	yes	no	yes	yes	yes	yes	no	no
Hsu 2022	Hsu (2022)	yes	yes	yes	yes	no	yes	yes	no
Marinovich 2023	Marinovich (2023)	yes	no	no	yes	yes	yes	yes	no
Riveira-Martin 2023	Riveira-Martin (2023)	yes	no	yes	yes	no	no	no	no
Kwon 2024	Kwon (2024)	yes	no	yes	yes	no	yes	no	no
Seker 2024	Seker (2024)	yes	no	no	yes	yes	yes	no	no
Larsen 2024	Larsen (2024)	yes	no	no	yes	yes	no	no	no
Hickman 2024	Hickman (2024)	yes	no	no	yes	yes	yes	yes	no
Kuhl 2024	Kuhl (2024)	yes	no	no	yes	yes	yes	no	no
Graham-Knight 2025	Graham-Knight (2025)	yes	no	yes	yes	yes	yes	no	no
Yamaguchi 2025	Yamaguchi (2025)	no	yes	yes	no	no	yes	no	no
Retson	Retson	yes	no	yes	yes	no	yes	no	no

2022	(2022)								
ScreenTrustCAD 2023	ScreenTrustCAD (2023)	yes	no	no	yes	no	no	yes	yes
MASAI 2025	MASAI (2025)	yes	no	no	yes	yes	no	yes	yes
AI-STREAM 2025	AI-STREAM (2025)	yes	no	no	yes	no	no	yes	yes
Lauritzen 2024	Lauritzen (2024)	yes	no	no	yes	no	no	yes	no
Elias-Cabot 2024	Elias-Cabot (2024)	yes	no	no	yes	no	no	no	no
Nepute 2025	Nepute (2025)	yes	no	no	yes	no	no	yes	no
Sandler Rahat 2025	Sandler Rahat (2025)	yes	no	no	yes	no	no	no	no
PRAIM 2025	PRAIM (2025)	yes	no	no	yes	no	no	yes	yes
Letter 2023	Letter (2023)	yes	no	no	yes	no	no	yes	no
McKinney 2020	McKinney (2020)	no	no	no	yes	no	yes	yes	no
