# Supplementary material for: Ten Years of Artificial Intelligence in Screening Mammography: A Systematic Review and Meta-Analysis of Diagnostic Accuracy and Clinical Implementation (Literature Published 2015–2025)
Source: Diagnostics (Basel). 2026 Sep 20;16(18):3045. doi: 10.3390/diagnostics16183045 (PMC13605948; doi:10.3390/diagnostics16183045)
Supplement: Supplementary file 1 [file diagnostics-16-03045-s001.zip › Supplementary_Materials/Tables/Table_S5_ongoing_trials.pdf]

Table S5. Registered evaluations of AI in mammographic screening

Identified by searching ClinicalTrials.gov on 11 August 2026 with the strategy in Table S1.3. Fifty-one registry records were retrieved; the table lists those enrolling at least 5,000 participants that evaluate a deep-learning system in a screening context. Two of these trials have reported and are included in the review. Of the remaining six, three were still recruiting and three had completed enrolment, and none had published results at the time of the search; none contributed data to the syntheses.

Registry ID	Country	Design	Planned enrolment	Start	Status	Primary outcome	Reported?
NCT06934239	United States	Randomised controlled trial	400,000	Oct 2025	Recruiting	Cancer detection rate; recall rate	No
NCT06032390	Norway	Randomised controlled trial	150,000	Nov 2024	Recruiting	Screen-detected breast cancers	No
NCT04838756 (MASAI)	Sweden	Randomised controlled trial	100,000	Apr 2021	Completed	Interval-cancer rate	Secondary outcomes reported (Lång 2023; Hernström 2025); primary endpoint pending
NCT06187350	Sweden	Prospective observational	60,000	Aug 2023	Completed	Cancer detection rate; positive predictive value	No
NCT04949776	Spain	Interventional, non-randomised	31,301	Mar 2022	Completed	Reading workload; detection rate	No
NCT05024591 (AI-STREAM)	South Korea	Prospective multicentre cohort	25,008	Feb 2021	Enrolment complete	Diagnostic accuracy with and without AI-CAD	Preliminary analysis reported (Chang 2025)
NCT07075679	Czechia	Randomised controlled trial	8,000	Oct 2025	Recruiting	Further-assessment rate	No
NCT05800132	France	Interventional, non-randomised	5,000	Jun 2023	Recruiting	Recall rate	No

What this tells us about publication bias

Two observations are relevant to the interpretation of the review.

First, the randomised evidence base is about to expand substantially. Three randomised trials totalling more than half a million women are in progress in three health systems that differ from Sweden in reading protocol, workforce and case mix. Any conclusion about the transferability of the MASAI result should be treated as provisional until at least one of them reports.

Second, two European programme evaluations that had completed enrolment, together covering more than 90,000 women, had not published results at the time of our search. We cannot know their direction. Their existence is one reason the non-randomised implementation tier of Analysis B, in which the pooled estimate exceeds the randomised one, is rated at very low certainty and flagged for suspected reporting bias in Table S3.

The interval-cancer endpoint of MASAI, which is the primary outcome of that trial and the outcome that would establish whether increased detection translates into benefit rather than overdiagnosis, remains in follow-up.
