# Supplementary material for: Ten Years of Artificial Intelligence in Screening Mammography: A Systematic Review and Meta-Analysis of Diagnostic Accuracy and Clinical Implementation (Literature Published 2015–2025)
Source: Diagnostics (Basel). 2026 Sep 20;16(18):3045. doi: 10.3390/diagnostics16183045 (PMC13605948; doi:10.3390/diagnostics16183045)
Supplement: Supplementary file 1 [file diagnostics-16-03045-s001.zip › Supplementary_Materials/Tables/Table_S6_PRISMA_2020_checklist.pdf]

Table S6. PRISMA 2020 checklist

Every item is marked as reported or not reported **as the revised manuscript now stands**. Items that the original submission described as planned rather than completed are flagged in the last column, because a reviewer correctly noted that the submitted checklist and the submitted manuscript disagreed on this point.

#	Item	Location in the revised manuscript	Status	Changed at revision?
1	Title identifies the report as a systematic review	Title	Reported	Date range clarified
2	Abstract: structured summary	Abstract	Reported	Rewritten
3	Rationale	Section 1, paragraphs 1–4	Reported	–
4	Objectives with an explicit statement of the question	Section 1, "Review question"; primary endpoints named	Reported	Endpoints now named explicitly
5	Eligibility criteria	Section 2.2	Reported	Grey literature and language criteria added
6	Information sources with the date each was last searched	Section 2.3; Table S1	Reported	Europe PMC, registries and citation searching added
7	Full search strategy for every database	Table S1	Reported	Was absent; now complete, and the strategy itself was corrected
8	Selection process, including how many reviewers and whether independently	Section 2.4; Section S1.6	Reported, with a measured agreement statistic	Third revision: the two-stage adjudication of all 250 discordant records is described accurately and published record by record
9	Data collection process	Section 2.5	Reported	Rewritten for clarity; selection rules added
10a	Outcomes sought and how results were selected	Section 2.5, rules 1 and 2; Section 2.7	Reported	New
10b	Other variables sought	Section 2.5	Reported	–
11	Risk-of-bias assessment: tool, how many reviewers, independence	Section 2.6; Table S2	Reported	Completed, not planned
12	Effect measures	Section 2.7, "Choice of outcome measure"	Reported	New, including the limits of AUC
13a	Processes for deciding which studies were eligible for each synthesis	Section 2.7, "Which studies entered which analysis"; Table 1	Reported	New

13b	Data preparation and conversions	Section 2.7	Reported	Logit transformation and Hanley–McNeil use now stated
13c	Tabulation and display methods	Figures 2–5; Tables 2a–2c	Reported	–
13d	Synthesis methods and model	Section 2.7	Reported	REML with Knapp–Hartung replaces DerSimonian–Laird
13e	Methods to explore heterogeneity	Section 2.7, "Exploring heterogeneity"	Reported	Subgroups and meta-regression added
13f	Sensitivity analyses	Section 2.7; Section 3.3	Reported	Estimator, leave-one-out and multi-system rules added
14	Assessment of risk of bias due to missing results	Section 2.7, "Small-study effects"; Figure S2	Reported	Egger test now possible at k = 13
15	Certainty assessment	Section 2.6; Table S3	Reported	Completed, not planned
16a	Study-selection results with a flow diagram	Section 3.1; Figure 1	Reported	Third revision: every arm of the flow now balances and the counts agree with Table 1
16b	Studies excluded at full text, with reasons	Figure 1; `fulltext_exclusions.csv` in Data S1	Reported	Third revision: all 133 exclusions listed report by report, and the Figure 1 tally is generated from that listing
17	Characteristics of each included study	Table 1	Reported	Rebuilt; "Examinations" column corrected
18	Risk of bias in each included study	Figure 5; Table S2	Reported	New
19	Results of individual studies	Tables 2a–2c; Figures 2 and 3	Reported	Restructured so each row compares like with like
20a	Studies contributing to each synthesis	Table 1, "Contribution" column	Reported	New
20b	Results of each synthesis with a measure of heterogeneity	Sections 3.3–3.6	Reported	–
20c	Investigations of causes of heterogeneity	Section 3.3, subgroups and meta-regression	Reported	New; this item was not met in the original submission
20d	Sensitivity analyses	Section 3.3	Reported	–
21	Risk of bias due to missing results	Section 3.3, final paragraph	Reported	–
22	Certainty of evidence for each outcome	Section 3.7; Table S3	Reported	New

23a	General interpretation	Section 4	Reported	Substantially rewritten and more cautious
23b	Limitations of the evidence	Section 4.1	Reported	Expanded
23c	Limitations of the review process	Section 4.1	Reported	Search and screening limitations now stated explicitly
23d	Implications	Section 4.2	Reported	Conditions for implementation now specified
24a	Registration and protocol	Section 2.1; Protocol S1	Reported	Protocol S1 now supplied
24b	Where the protocol can be accessed	Section 2.1	Reported	–
24c	Amendments to the protocol	Section 2.9	Reported	Second revision: amendments separated into those made after the first and after the second round of review, and the word "pre-specified" removed where it did not apply
25	Support and funding	Funding statement	Reported	–
26	Competing interests	Conflicts of Interest	Reported	–
27	Availability of data, code and other materials	Data Availability Statement; Data S1	Reported	Code and logs now published

Item 8, stated plainly. Title-and-abstract screening of 1,743 records was performed by a written, published rule set rather than by two independent human reviewers. At the second revision that stage was verified against an independently specified second screening procedure applied to the same records: agreement was moderate (Cohen's κ 0.47, 95% CI 0.41–0.53), and three eligible studies the first pass had missed were added to the review. All 250 discordant records carry a final decision and a documented reason: 230 were adjudicated at record level against the eligibility criteria and the 20 with any route to inclusion were retrieved in full and adjudicated by two reviewers. The first of those two stages is a single documented assessment, not duplicate independent human screening. Section S1.6 reports the comparison and the adjudication in full, and Section 4.1 of the manuscript states what it implies. For the full-text stage, assessment of all 157 reports that reached it was performed independently in duplicate with a third reviewer for disagreements, but those judgements were reconciled to consensus as the assessment proceeded rather than recorded separately first, so no agreement statistic can be computed for that stage after the fact.
