# Supplementary material for: Ten Years of Artificial Intelligence in Screening Mammography: A Systematic Review and Meta-Analysis of Diagnostic Accuracy and Clinical Implementation (Literature Published 2015–2025)
Source: Diagnostics (Basel). 2026 Sep 20;16(18):3045. doi: 10.3390/diagnostics16183045 (PMC13605948; doi:10.3390/diagnostics16183045)
Supplement: Supplementary file 1 [file diagnostics-16-03045-s001.zip › Supplementary_Materials/Tables/Table_S7_PRISMA_DTA_checklist.pdf]

Table S7. PRISMA-DTA checklist

#	Item	Location	Status
1	Title identifies the report as a systematic review of diagnostic test accuracy	Title	Reported
2	Structured abstract covering background, methods, results and conclusions	Abstract	Reported
D3	Abstract states the index test, the reference standard and the target condition	Abstract, Methods sentence	Reported
3	Rationale in the context of what is already known	Section 1	Reported
D4	Rationale states the intended use and clinical role of the index test	Section 1, "Review question"; Section 4.2	Reported
4	Objectives with reference to participants, index test and target condition	Section 1, PIRD statement	Reported
5	Eligibility criteria including the reference standard and target condition	Section 2.2	Reported
6	Information sources and the date each was last searched	Section 2.3; Table S1	Reported
7	Full search strategy for at least one database, repeatable	Table S1, all four sources	Reported
8	Study selection process	Section 2.4; Section S1.6	Reported, with an agreement statistic for the verified first stage
9	Data collection process	Section 2.5	Reported
10	Definitions used in data extraction, including for the target condition	Sections 2.2 and 2.5	Reported
D11	Rationale for a priori subgroups and investigations of heterogeneity	Section 2.7, "Exploring heterogeneity"	Reported
D12	Definitions of test positivity and any thresholds	Section 2.7; Table 2b, per study	Reported
12	Risk-of-bias assessment method	Section 2.6; Table S2	Reported

13	Diagnostic accuracy measures used	Section 2.7, "Choice of outcome measure"	Reported
14	Synthesis methods, model and software	Section 2.7; code published as Data S1	Reported
15	Additional analyses	Sections 2.7 and 2.9	Reported
16	Study selection results with a flow diagram	Figure 1	Reported
17	Characteristics of included studies	Table 1	Reported
18	Risk of bias within studies	Figure 5; Table S2	Reported
D19	Results for each study: 2 × 2 data or accuracy measures with a precision estimate	Tables 2a and 2b; Table S4b gives reconstructed 2 × 2 cells	Reported
20	Results of syntheses	Sections 3.3–3.6; Figures 2 and 3	Reported
21	Risk of bias across studies	Section 3.3, final paragraph; Figure S2	Reported
22	Additional analyses	Section 3.3	Reported
23	Summary of evidence, with the strength and limitations of each main outcome	Section 4; Table S3	Reported
24	Limitations, including risk of bias and issues of applicability	Section 4.1	Reported
25	Conclusions and implications for practice	Sections 4.2 and 5	Reported
D26	Implications for the clinical role of the index test	Section 4.2	Reported
27	Funding and role of funders; protocol registration	Funding; Section 2.1	Reported

Notes on three items that needed particular attention

D12, thresholds. Every study in the bivariate synthesis reports a single operating point, but the rule used to select it differs between studies. Table 2b prints the rule beside each estimate rather than presenting the nine thresholds as though they were interchangeable. Two studies optimised the Youden index on the same data used for evaluation, which inflates both measures, and this is stated where the result is reported rather than only in a footnote.

D19, 2 × 2 data. Six of the nine studies in Analysis A2 did not print a 2 × 2 table. Cells were reconstructed from the reported sensitivity, specificity and denominators, and the reconstructed values are published in Table S4b so that a reader can check the arithmetic.

14, software. The synthesis was implemented in Python. The full script is published, and re-running it regenerates every estimate in the manuscript from the extraction tables. At a reviewer's request a parallel implementation using `metafor` and `mada` is also supplied, clearly labelled as an unexecuted reference implementation, because R was not available in the environment in which the review was conducted.
