# Supplementary material for: Ten Years of Artificial Intelligence in Screening Mammography: A Systematic Review and Meta-Analysis of Diagnostic Accuracy and Clinical Implementation (Literature Published 2015–2025)
Source: Diagnostics (Basel). 2026 Sep 20;16(18):3045. doi: 10.3390/diagnostics16183045 (PMC13605948; doi:10.3390/diagnostics16183045)
Supplement: Supplementary file 1 [file diagnostics-16-03045-s001.zip › Supplementary_Materials/Tables/Table_S8_extraction_template.pdf]

Table S8. Data-extraction template

The blank form used by both extractors. One form was completed per study; where a study reported several AI systems or several cohorts, one form was completed per system and per cohort, and the selection rules in Section 2.5 of the manuscript then determined which entered the main analysis.

Part 1 — Identification

Field	Entry	Notes
Study label (first author, year)		
PubMed identifier		
DOI		
Additional reports of the same study		Give identifiers; the study is counted once
Country		
Screening programme or data source		
Enrolment period (from, to)		
Funding source and vendor involvement		Record whether the AI vendor funded or co-authored

Part 2 — Design and population

Field	Entry	Options
Design		MRMC reader study / retrospective case-control / retrospective consecutive cohort / external validation / prospective paired-reader / before-and-after cohort / randomised trial
Sampling		Consecutive / random / enriched case-control
Modality		DM / DBT / both
Setting		Screening only / screening and diagnostic mixed
Number of screening examinations analysed		
Number of women		If different from examinations
Number of cancers		
Number of controls or cancer-free		

examinations		
Mean or median age		
Breast-density distribution reported?		Yes / No
Ethnicity distribution reported?		Yes / No

Part 3 — Index test

Field	Entry	Notes
AI system name and vendor		
Version		Record "not reported" rather than leaving blank
Architecture		Record "not reported" rather than inferring
Was the system evaluated on data independent of its development?		Yes / No / Cannot tell
Role of AI in the workflow		Standalone / triage / second reader / reader replacement / concurrent support
Output scale		
Threshold used		Give the value
**Rule used to select the threshold**		Vendor default / matched to a human reader's sensitivity / matched to a human reader's specificity / optimised on the evaluation data / other
If more than one system was evaluated, list all with their results		The median-performing system enters the main analysis

Part 4 — Reference standard and comparator

Field	Entry	Notes
Reference standard		
Follow-up window for negatives		Months
Were interval cancers included in the target condition?		Yes / No
Ascertainment method		Registry linkage / clinical follow-up / other
Comparator		Single reader / double reading / double reading with arbitration / no comparator

Was the comparator measured in the same population?		Yes / No

Part 5 — Outcomes

Field	AI arm	Comparator arm
AUC (95% CI)		
Sensitivity % (n/N)		
Specificity % (n/N)		
True positives		
False negatives		
False positives		
True negatives		
Cancer detection rate per 1000		
Recall rate %		
Positive predictive value of recall %		
Interval cancer rate		
Screen-reading workload change %		
Reading time		

Part 6 — Appraisal

Field	Entry
QUADAS-2: patient selection, risk of bias	Low / Unclear / High
QUADAS-2: index test, risk of bias	Low / Unclear / High
QUADAS-2: reference standard, risk of bias	Low / Unclear / High
QUADAS-2: flow and timing, risk of bias	Low / Unclear / High
QUADAS-2: patient selection, applicability	Low / Unclear / High
QUADAS-2: index test, applicability	Low / Unclear / High
QUADAS-2: reference standard, applicability	Low / Unclear / High
Written rationale	Free text; required for every non-Low judgement
Cohort overlap with another included study?	Yes (name it) / No

Part 7 — Synthesis eligibility

Analysis	Eligible?	If not, why
A — standalone AUC	Yes / No	
A2 — bivariate sensitivity and specificity	Yes / No	
B — cancer detection rate ratio	Yes / No	
C — recall rate ratio	Yes / No	

Reconciliation

Field	Entry
Extractor 1	
Extractor 2	
Fields with a discrepancy	
How each was resolved	
Third reviewer consulted?	Yes / No
